# *Plasmodium falciparum* S-Adenosylmethionine Synthetase Is Essential for Parasite Survival through a Complex Interaction Network with Cytoplasmic and Nuclear Proteins

**DOI:** 10.3390/microorganisms10071419

**Published:** 2022-07-14

**Authors:** Jean Pierre Musabyimana, Ute Distler, Juliane Sassmannshausen, Christina Berks, Janice Manti, Sandra Bennink, Lea Blaschke, Paul-Christian Burda, Ansgar Flammersfeld, Stefan Tenzer, Che Julius Ngwa, Gabriele Pradel

**Affiliations:** 1 Division of Cellular and Applied Infection Biology, Institute of Zoology, RWTH Aachen University, Worringerweg 1, 52074 Aachen, Germany; musabyimana@bio2.rwth-aachen.de (J.P.M.); sassmannshausen@bio2.rwth-aachen.de (J.S.); christina.berks@rwth-aachen.de (C.B.); janice.manti@rwth-aachen.de (J.M.); bennink@bio2.rwth-aachen.de (S.B.); lea.blaschke@rwth-aachen.de (L.B.); ansgar.flammersfeld@rwth-aachen.de (A.F.); ngwa.che@bio2.rwth-aachen.de (C.J.N.); 2 Proteomics Core Facility, Institute of Immunology, University Medical Center of the Johannes-Gutenberg University Mainz, Langenbeckstraße 1, 55131 Mainz, Germany; ute.distler@uni-mainz.de (U.D.); tenzer@uni-mainz.de (S.T.); 3 Centre for Structural Systems Biology (CSSB) c/o DESY, Bernhard Nocht Institute, University of Hamburg, Notkestraße 85, Building 15, 22607 Hamburg, Germany; burda@bnitm.de

**Keywords:** *Plasmodium falciparum*, malaria, histone methylation, polyamine biosynthesis, SAMS, SAM, interactome, transcriptional regulation, proteasome, drug target

## Abstract

S-adenosylmethionine synthetase (SAMS) is a key enzyme for the synthesis of the lone methyl donor S-adenosyl methionine (SAM), which is involved in transmethylation reactions and hence required for cellular processes such as DNA, RNA, and histone methylation, but also polyamine biosynthesis and proteostasis. In the human malaria parasite *Plasmodium falciparum*, *Pf*SAMS is encoded by a single gene and has been suggested to be crucial for malaria pathogenesis and transmission; however, to date, *Pf*SAMS has not been fully characterized. To gain deeper insight into the function of *Pf*SAMS, we generated a conditional gene knockdown (KD) using the *glmS* ribozyme system. We show that *Pf*SAMS localizes to the cytoplasm and the nucleus of blood-stage parasites. *Pf*SAMS-KD results in reduced histone methylation and leads to impaired intraerythrocytic growth and gametocyte development. To further determine the interaction network of *Pf*SAMS, we performed a proximity-dependent biotin identification analysis. We identified a complex network of 1114 proteins involved in biological processes such as cell cycle control and DNA replication, or transcription, but also in phosphatidylcholine and polyamine biosynthesis and proteasome regulation. Our findings highlight the diverse roles of *Pf*SAMS during intraerythrocytic growth and sexual stage development and emphasize that *Pf*SAMS is a potential drug target.

## 1. Introduction

The S-adenosylmethionine synthetase SAMS, also called methionine adenosyltransferase (MAT), is an important enzyme found in all living organisms that catalyzes the synthesis of S-adenosyl methionine (SAM) from methionine and adenosine triphosphate (ATP). SAM is the sole methyl donor in cellular processes such as the methylation of histones, DNA, and RNA, which are important in the regulation of transcription and translation [1]. SAM is not only required for methylation events but also for transsulfuration, polyamine biosynthesis, and proteostasis, with importance for cell growth, survival, and proliferation [2,3]. Due to the diverse functions of SAM, the inhibition of the enzyme SAMS results in significant consequences for the cell; e.g., MAT enzyme deregulation in humans results in several types of cancers, such as leukemia and hepatocellular carcinoma [4,5,6]. For these reasons, SAMS has been regarded as a drug target, and novel inhibitors are being designed to target the enzyme [7,8].

In recent years, the crucial role of SAM and SAMS in human malaria has been acknowledged. Malaria accounted for over 241 million infections and 627,000 deaths in 2020 [9], with *P. falciparum*, the causative agent of malaria tropica, being the most serious form. The life cycle progression of *P. falciparum* in the human, as well as the mosquito vector, requires tight regulation of various cellular processes, including transcriptional and translational regulation, or protein turnover control, but also membrane biosynthesis and modification. *Pf*SAMS is encoded by a single gene, and its functionality and druggability have previously been shown [10,11].

Recent studies particularly associated *Pf*SAMS with histone methylation events, catalyzed by histone methyl transferases which transfer methyl groups from SAM to histones. Histone methylation is crucial for the regulation of gene expression in both the asexual blood stages and the sexual stages of the parasite. These methylation events play a major role, for example, during the expression of virulence-associated clonally variant multigene families, such as the 60 *var* genes encoding the *P. falciparum* erythrocyte membrane protein *Pf*EMP1 [12,13,14,15,16,17,18,19]. The switch of *var* expression and thus *Pf*EMP1 structure alters the antigenic pattern of the infected red blood cells (infected RBCs; iRBCs) and, in consequence, pathogenesis of the tropical disease. The expression of *var* genes relies on epigenetic mechanisms that induce dynamic changes in the chromatin structure. Only the active *var* gene copy assumes a euchromatic state, characterized, among others, by the tri-methylated lysine 4 and acetyl lysine 9 of histone H3 (H3K4me3 and H3K9ac, respectively) [12,20,21]. On the other hand, *var* gene silencing is linked to H3K9 and H3K36 tri-methylation (H3K9me3, H3K36me3) [13,22,23,24,25,26].

Histone methylation is also important in regulating sexual commitment, a process in which the asexual blood-stage parasites enter the sexual pathway to form gametocytes, which, in consequence, enables parasite transmission from the human to the mosquito vector [27,28,29]. Sexual commitment is promoted by environmental stress signals, particularly low serum levels of lysophosphatidylcholine (lysoPC) needed by the parasite to synthesize phosphatidylcholine (PC) [30,31,32]. Sexual commitment is closely linked to the plasmodial heterochromatin protein HP1. This regulator specifically binds to the histone H3 methylation mark H3K9me3 to maintain the heterochromatin state. HP1 binding suppresses sexual commitment in *P. falciparum* by silencing the gene encoding the transcription factor AP2-G, a member of the apicomplexan Apetala2/ethylene response factor (AP2/ERF) DNA-binding protein family [33,34,35,36,37]. During sexual commitment, the ap2-g locus is activated by HP1 release, a process promoted by the modulator GDV-1 (gametocyte development protein 1) [38,39,40]. Once AP2-G is synthesized, it initiates the expression of various early gametocyte genes, e.g., the synthesis of other AP2 transcription factors such as the female-specific AP2-FG [41,42].

Recently, the potential link between nutrient availability and epigenetic regulation of sexual commitment has been investigated in detail. It was shown that lysoPC deficiency and impaired PC synthesis initiate the transcriptional upregulation of early gametocyte-specific genes, particularly the *ap2-g* gene, while high PC levels counteract gametocyte induction [30,32]. If lysoPC is available, PC is generated from precursors by the *de novo* cytidine diphosphate (CDP)-choline (Kennedy) pathway, but in its absence, PC is synthesized by triple-methylation of phosphoethanolamine using ethanolamine (from serum) or serine (from hemoglobin) as external precursors [43]. This pathway involves the activity of the phosphoethanolamine N-methyltransferase *Pf*PMT that catalyzes the conversion of phosphoethanolamine into phosphocholine to compensate for the lack of PC precursors. In accord, parasite lines deficient in *Pf*PMT die in serum lacking these precursors [31]. This alternative route of PC synthesis via the *Pf*PMT pathway is upregulated during the sexual commitment of lysoPC-depleted parasites, which among others, is reflected by increased transcript synthesis of *Pf*PMT and of *Pf*SAMS [30]. It has been postulated that under reduced lysoPC conditions, the parasites need to utilize SAM to generate PC, and, in consequence, less SAM could be used to repress the *ap2-g* locus via histone methylation [44]. In fact, a new report [45] confirmed that lack of lysoPC in the medium results in increased *Pf*PMT expression and decreased SAM levels in *P. falciparum*, while *Pf*PMT deficiencies increase intracellular SAM levels and repress sexual commitment.

Apart from histones, the methylation of other molecules requiring SAM as a methyl donor has been reported in *P. falciparum*, such as the methylation of DNA, RNA, or non-histone proteins [46,47,48,49]. Another process in which *Pf*SAMS is potentially involved is polyamine biosynthesis. In eukaryotes, polyamine synthesis begins with ornithine, which is a product of the urea cycle. Its decarboxylation leads to polyamine putrescine, which is then used for the generation of spermidine and spermine, and the synthesis of both molecules requires SAM-dependent transmethylation. Polyamines facilitate proliferative processes by modifying the chromosomal structure or influencing protein-DNA interactions [50]. Polyamine biosynthesis pathways have been identified in the malaria parasite *P. falciparum* [51], and there is some evidence of a dependency between the polyamine pathway and intraerythrocytic development [52,53,54].

The above-mentioned studies point to the major roles of SAM in cellular processes and hence the viability of *P. falciparum*. However, *Pf*SAMS, the enzyme catalyzing the synthesis of SAM, is, to date, not well characterized. In this study, we shed light on the function of *Pf*SAMS in the *P. falciparum* blood stages and unveil the interactome of the enzyme.

## 2. Materials and Methods

### 2.1. Gene Identifiers

The following PlasmoDB gene identifiers (gene IDs) (www.plasmodb.org; [55] accessed on 1 May 2022) are assigned to the genes and gene products investigated in this study: *Pf*39 [PF3D7_1108600]; *Pf*92 [PF3D7_1364100]; *Pf*s230 [PF3D7_0209000]; *Pf*Aldolase [PF3D7_1444800]; *Pf*AMA1 [PF3D7_1133400]; *Pf*Falcilysin [PF3D7_1360800]; *Pf*FNPA [PF3D7_1451600; synonym: LAP5]; histone H3 [PF3D7_0610400]; *Pf*SAMS [PF3D7_0922200].

### 2.2. Primary Antibodies

The following primary antibodies and antisera were used: rat anti-HA antibody (Roche, Basel, Switzerland); mouse anti-GFP antibody (Roche); mouse polyclonal antisera against *Pf*39 [56]; rabbit polyclonal antisera against *Pf*s230 [57]; mouse polyclonal antisera against *Pf*Falcilysin [58]; rabbit anti-H3K4me3 antibody (abcam, Cambridge, UK), rabbit anti-H3K18me1 antibody (AB clonal, Cummings Park, W). The generation of polyclonal mouse antisera against *Pf*92 is described below.

### 2.3. Parasite Culture

The gametocyte-producing strain *P. falciparum* NF54 (WT NF54) was cultivated in vitro in RPMI 1640/ HEPES medium (Gibco) supplemented with 10% *v*/*v* heat-inactivated human serum and A^+^ erythrocytes at 5% *v*/*v* hematocrit. The medium was completed with 50 µg/mL hypoxanthine (Sigma-Aldrich, Taufkirchen, Germany), and 10 µg/mL gentamicin (Gibco) and cultures were grown in an atmosphere of 5% CO_2_, 5% O_2_, and 90% N_2_ at a constant temperature of 37 °C. For cultivation of the *Pf*SAMS-HA-KD line, the selection drug WR99210 (Jacobus Pharmaceutical Company, Princeton, USA was added in a final concentration of 4 nM. For knockdown of *Pf*SAMS, the *Pf*SAMS-HA-KD cultures were incubated with a complete medium supplemented with 5 mM glucosamine hydrochloride (GlcN; D-(+)-glucosamine hydrochloride; Sigma-Aldrich, Taufkirchen, Germany) for 72 or 120 h. Human serum and erythrocyte concentrate were obtained from the Department of Transfusion Medicine, University Hospital Aachen, Germany. Donor sera and blood samples were pooled and kept anonymous. The work with human blood was approved by the Ethics Commission of the RWTH University Hospital (EK 007/13). To synchronize the asexual parasite blood stages, parasite cultures with 3–4% ring stages were centrifuged, the pellet was resuspended in 5× pellet’s volume of 5% *w*/*v* sorbitol (AppliChem)/ddH_2_O and incubated for 10 min at room temperature (RT). Cells were washed once with RPMI to remove the sorbitol and further cultivated as described above. Schizonts and gametocytes were enriched via Percoll gradient centrifugation (GE Healthcare Life Sciences, Chicago, IL, USA) as described previously [59].

### 2.4. Generation of Mouse Anti-Pf92 Antisera

A recombinant protein corresponding to *Pf*92 (spanning aa 589–762) was expressed as a fusion protein with an N-terminal maltose binding protein (MBP)-tag using the pMAL^TM^c5x-vector (New England Biolabs, Ipswich, MA, USA). Cloning was mediated by the addition of the restriction sites XmnI/PstI to the ends of gene fragments that were PCR-amplified from WT NF54 gDNA, using *Pf*92rp1 forward primer and *Pf*92rp1 reverse primer (for primer sequences, see Table S1). Recombinant proteins were expressed using *E. coli* BL21 (DE3) RIL according to the manufacturer’s protocol (Stratagene, San Diego, CA, USA). The recombinant fusion protein was purified via affinity chromatography from bacterial extracts using amylose resin (New England Biolabs, Frankfurt am Main, Germany) according to the manufacturer’s protocol, followed by PBS buffer exchange via filter centrifugation using Amicon Ultra 15 (Sigma-Aldrich, Taufkirchen, Germany) according to the manufacturer’s protocol. Protein concentrations were determined via Bradford assay, following the standard protocol. Immune sera were generated by immunization of 6-week-old NMRI mice (Charles River Laboratories, Wilmington, DC, USA) via subcutaneous injection of 100 µg recombinant protein emulsified in Freund’s incomplete adjuvant (Sigma-Aldrich, Taufkirchen, Germany) followed by a boost after 4 weeks with 50 µg of recombinant protein. Mice were anesthetized 10 days after the boost by intraperitoneal injection of a ketamine–xylazine mixture according to the manufacturer’s protocol (Sigma-Aldrich, Taufkirchen, Germany). Polyclonal immune sera were collected via heart puncture and pooled from three mice immunized with the same antigen. NMS were collected for negative control in the experiments. Experiments for the generation of antisera in mice were approved by the animal welfare committee of the District Council of Cologne, Germany (ref. no. 84-02.05.30.12.097 TVA).

### 2.5. Generation of the PfSAMS-HA-KD Parasite Line

The *Pf*SAMS-HA-KD parasite line was generated via single cross-over homologous recombination using the pSLI-HA-*glmS* vector (kindly provided by Dr. Ron Dzikowski, the Hebrew University of Jerusalem). An 1155-bp gene fragment homologous to the C-terminal part of the *pfsams* gene was amplified using the *Pf*SAMS-HA-KD forward primer *Pf*SAMS-HA-*glmS* NotI FP and the *Pf*SAMS-HA-KD reverse primer *Pf*SAMS-HA-*glmS* XmaI RP (for primer sequences, see Table S1). The stop codon was excluded from the homologous gene fragment. Ligation of the insert with the vector backbone was mediated by NotI and XmaI restriction sites. A WT NF54 culture synchronized for 5% ring stages was loaded with 100 µg vector in transfection buffer via electroporation (310 V, 950 µF, 10 ms; Bio-Rad gene-pulser) as described [60,61]. For the selection of parasites carrying the vector, WR99210 was added to a final concentration of 4 nM, and successful integration of the vector was confirmed by diagnostic integration PCR using pSLI-*Pf*SAMS 5′Int *glmS* FP (1), pSLI-*Pf*SAMS 3’Int *glmS* RP (2), pARL-HA-*glmS* FP (3) and pSLI-HA-*glmS* RP (4) (for primer location, see Figure S1A; for primer sequences, see Table S1). To select integrants, neomycin (G418; Sigma-Aldrich) was added to the culture at a final concentration of 550 μg/mL until the parasites disappeared (approximately two weeks). During this time, the culture was fed daily. Successful selection of integrants was monitored by diagnostic PCR as described above.

### 2.6. Generation of the PfSAMS-GFP-BirA Parasite Lines

A sequence encoding for the green fluorescent protein (GFP) was amplified from the pSLI-TGD-GFP vector [62], using primers GFP FP and GFP RP. The sequence encoding BirA was amplified from a plasmid containing the respective gene, which was codon-optimized for expression in *P. falciparum* [63], using primers BirA FP and BirA RP (for primer sequences, see Table S1). Both PCR products were introduced in a GIBSON reaction into a KpnI and AvrII-digested pARL-*pfama1*-GFP vector [64], resulting in pARL-GFP-BirA. To exchange the asexual blood stage-specific *pfama1* promotor [65] with the gametocyte-specific promotor of *pffnpa* [66], the *pfama1* promotor was exchanged by NotI/KpnI restriction digestion. A 685-bp fragment of the 5′ *pffnpa* promotor region was amplified using the *pffpna*-5′ FP and the *pffnpa*-5′ RP (for primer sequences, see Table S1), and ligation into the vector backbone was mediated by NotI/KpnI. The *Pf*SAMS-*pfama1*-GFP-BirA and *Pf*SAMS-*pffnpa*-GFP-BirA parasite lines were generated using the respective vectors (Figure S4A). The *pfsams* full-length gene was amplified from WT NF54 gDNA using the two primer pairs *Pf*SAMS-*pffnpa*-GFP-BirA FP KpnI/*Pf*SAMS-*pffnpa*-GFP-BirA RP AvrII and *Pf*SAMS-*pfama1*-GFP-BirA ApaI FP/*Pf*SAMS-*pfama1*-GFP-BirA AvrII RP (for primer sequences, see Table S1). The stop codon was excluded from the full-length gene sequence. Ligation of the insert with the vector backbones was mediated by KpnI/AvrII and ApaI/AvrII. The plasmid was sequenced to confirm that the encoding segment was inserted in frame with the GFP-encoding sequence. Transfection of parasites was performed as described above. For the selection of parasites carrying the vectors, WR99210 was added to a final concentration of 4 nM, and successful uptake of the vector was confirmed by diagnostic PCR using the two primer pairs *Pf*SAMS-*pfama1*-GFP-BirA ApaI FP/GFP RP and *Pf*SAMS-*pffnpa*-GFP-BirA KpnI FP/GFP RP. As a control, the aldolase gene was amplified using the primer pair Aldolase RT FP/Aldolase RT RP (for primer sequences, see Table S1; for primer location, see Figure S4A).

### 2.7. Indirect Immunofluorescence Assay

Mixed asexual blood-stage and gametocyte cultures of the *Pf*SAMS-HA-KD, the *Pf*SAMS-*pfama1*-GFP-BirA, and the *Pf*SAMS-*pffnpa*-GFP-BirA lines were air-dried as cell monolayers on glass slides and subsequently fixed in a methanol bath at −80 °C for 10 min. For membrane permeabilization and blocking of non-specific binding, fixed cells were sequentially incubated in 0.01% *w*/*v* saponin/0.5% *w*/*v* BSA/PBS and 1% *v*/*v* neutral goat serum (Sigma-Aldrich)/PBS for 30 min at RT. After blocking, the preparations were incubated with rat anti-HA or mouse anti-GFP antibody, diluted in 0.5% *w*/*v* BSA/PBS for 2 h at 37 °C. Following washing, binding of the primary antibody was detected by incubation with Alexa Fluor 488-conjugated goat anti-rat or anti-mouse secondary antibody (Thermo Fisher Scientific, Waltham, MA, USA). To immunolabel biotinylated proteins, Alexa Fluor 594-conjugated streptavidin was used (Thermo Fisher Scientific, Waltham, MA, USA). Mouse antisera directed against *Pf*39 or *Pf*92 were used to highlight the asexual blood stages, and rabbit antisera directed against *Pf*s230 were used to highlight gametocytes, followed by incubation with polyclonal Alexa Fluor 488- or 594-conjugated goat anti-mouse or anti-rabbit secondary antibodies (Invitrogen Molecular Probes; Eugene, OR, USA). Alternatively, the asexual blood stages were stained with 0.01% *w*/*v* Evans Blue (Sigma-Aldrich; Taufkirchen, Germany)/PBS for 3 min at RT followed by 5 min washing with PBS. The parasite nuclei were highlighted by treatment with Hoechst 33342 nuclear stain (Invitrogen) for 1 min at RT. Cells were washed with PBS, mounted with anti-fading solution AF2 (CitiFluor^TM^, Hatfield, PA, USA), and sealed with nail polish. Specimen were examined with a Leica DM 5500 B microscope, and digital images were processed using the Adobe Photoshop CS software.

### 2.8. Subcellular Fractioning

To determine the subcellular localization, protein extraction was carried out using a series of lysis and extraction buffers. In brief, ~3 × 10^6^ schizonts of the *Pf*SAMS-HA-KD line were purified via Percoll gradient and liberated from the RBCs with 0.03% *w*/*v* saponin/PBS for 3 min at 4 °C. After washing with PBS, parasites were hypotonically lysed in 100–200 µL of the lysis buffer containing 20 mM HEPES, 10 mM KCl, 1 mM EDTA, 1 mM dithiothreitol (DTT), 1 mM PMSF, 1% Triton X-100 (pH 7.8), supplemented with protease inhibitor cocktail (complete EDTA-free, Roche) for 10 min on ice. Following centrifugation at 2500× *g* for 5 min at 4 °C, the cytosolic proteins were harvested with the supernatant and stored at −80 °C. After several steps of washing the remaining pellet with the lysis buffer, nuclear proteins were extracted with about 2 volumes of the extraction buffer containing 20 mM HEPES, 800 mM KCl, 1 mM EDTA, 1 mM DTT, and 1 mM PMSF (pH 7.8), supplemented with protease inhibitor cocktail. Supernatants were centrifuged to remove residual material. The individual fractions were subjected to Western blotting as described below.

### 2.9. Western Blotting

Asexual blood-stage parasites of the WT NF54, the *Pf*SAMS-HA-KD line, the *Pf*SAMS-*pfama1*-GFP-BirA, and the *Pf*SAMS-*pffnpa*-GFP-BirA lines were harvested from mixed or synchronized cultures, while gametocytes were enriched by Percoll purification. Parasites were released from iRBCs with 0.015% *w*/*v* saponin/PBS for 10 min at 4 °C, washed with PBS, and resuspended in lysis buffer (0.5% Triton X-100, 4% *w*/*v* SDS, 0.5xPBS) supplemented with protease inhibitor cocktail; 5x SDS-PAGE loading buffer containing 25 mM DTT was added to the lysates, samples were heat-denatured for 10 min at 95 °C and separated via SDS-PAGE. Following gel electrophoresis, separated parasite proteins were transferred to Hybond ECL nitrocellulose membrane (Amersham Biosciences, Buckinhamshire, UK) according to the manufacturer’s protocol. Non-specific binding was blocked by incubation of the membranes in Tris-buffered saline containing 5% *w*/*v* skim milk, pH 7.5, followed by immune recognition overnight at 4 °C using polyclonal mouse anti-*Pf*39 antisera, mouse anti-GFP antibody, or rat anti-HA antibody. After washing, membranes were incubated with the respective alkaline phosphatase-conjugated goat secondary antibody (Sigma-Aldrich) for 1 h at RT. Biotinylated proteins were directly labeled using alkaline phosphatase-coupled streptavidin (Sigma-Aldrich). The blots were developed in a solution of nitroblue tetrazolium chloride (NBT) and 5-brom-4-chlor-3-indoxylphosphate (BCIP; Merck, Darmstadt, Germany) for 5–30 min at RT. Blots were scanned and processed using the Adobe Photoshop CS software. Band intensities were measured using the ImageJ program version 1.51f.

### 2.10. Asexual Blood Stage Replication Assay

To compare asexual blood-stage replication between the GlcN-treated and untreated *Pf*SAMS-HA-KD lines, synchronized asexual blood-stage cultures were set to an initial parasitemia of 0.25% ring stages and cultivated in a complete medium. The expression of *Pf*SAMS was knocked down by treatment of the culture with GlcN at a final concentration of 5 mM. The GlcN-treated culture was compared to the untreated culture cultivated in a normal cell culture medium. The parental WT NF54 treated with GlcN and untreated was used as negative controls. Giemsa-stained thin blood smears were prepared every 24 h over a time period of 72 h at four different time points (0, 24, 48, and 72 h post-seeding). The parasitemia of each time point was determined microscopically at 1000-fold magnification by counting the percentage of parasites in 1000 RBCs. To identify the blood stages (ring, trophozoites, schizonts) present in the cultures at a given time point, 100 iRBCs were counted per setting. For each assay, three experiments were performed, each in triplicate. Data analysis was performed using MS Excel 2016 and GraphPad Prism 5.

### 2.11. Gametocyte Development Assay

To compare gametocyte development between the GlcN-treated and untreated *Pf*SAMS-HA-KD line, tightly synchronized schizont stage cultures were set to a parasitemia of 0.5% with 5% hematocrit and cultivated in a cell culture medium at 37 °C for 72 h. The expression of *Pf*SAMS was knocked down by treatment of the culture with GlcN at a final concentration of 5 mM. The untreated and GlcN-treated parental WT NF54 was used as negative control. The parasitemia was determined in each culture, and the cultures were adjusted to a parasitemia of 5%. Gametocytogenesis was induced by the addition of lysed RBCs for 24 h [61,67]. Cells were washed, and cultures were maintained in a cell culture medium supplemented with heparin at a final concentration of 20 U/mL for 4 d to kill the asexual blood stages, followed by cultivation in a normal cell culture medium. Cultures were maintained in a cell culture medium containing GlcN over a time period of 7 d, and samples were taken at days 3, 5, and 7 post-gametocyte induction for Giemsa smear preparation. Gametocytemia was determined per 1000 RBCs. For this assay, one experiment was performed, and gametocytemia was measured in triplicate. Data analysis was performed using MS Excel 2016 and GraphPad Prism 5.

### 2.12. Preparation of Samples for BioID Analysis

Biotinylation by the BirA ligase was induced by adding biotin in a final concentration of 50 µM to the corresponding parasite culture for 20–24 h. Cells were washed twice with RPMI incomplete medium, and schizonts or gametocytes were enriched via Percoll gradient centrifugation. The parasites were resuspended in 100 µL binding buffer (Tris-buffered saline containing 1% Triton X-100 and protease inhibitor), and the sample was sonicated on ice (2 × 60 pulses at 30% duty cycle). After the addition of another 100 µL cold Tris-buffered saline, one more session of sonication was performed. After centrifugation (5 min, 16,000× *g*, 4 °C), the supernatant was transferred to a new reaction tube and mixed with 100 µL pre-equilibrated Cytiva Streptavidin Mag Sepharose™ Magnet-Beads (Fisher Scientific). The sample was incubated with slow end-over-end mixing at 4 °C overnight. After six washing steps with 3× RIPA buffer containing 0.03% SDS and three times with 25 mM Tris buffer (pH 7.5), biotinylated proteins were eluted from the beads by the addition of 40 µL of 1% *w*/*v* SDS/5 mM biotin in Tris buffer (pH 7.5) and incubation at 95 °C for 5 min.

### 2.13. Proteolytic Digestion

Samples were processed by single-pot solid-phase-enhanced sample preparation (SP3) as described [68,69]. In brief, proteins bound to the streptavidin beads were released by incubating the samples for 5 min at 95° in an SDS-containing buffer (1% (*w*/*v*) SDS, 5 mM biotin in water/Tris, pH 8.0), as described above. After elution, proteins were reduced and alkylated using DTT and iodoacetamide (IAA), respectively. Afterward, 2 µL of carboxylate-modified paramagnetic beads (Sera-Mag Speed Beads, GE Healthcare, Chicago, USA, 0.5 μg solids/μL in water as described [68]) were added to the samples. After adding acetonitrile to a final concentration of 70% *v*/*v*, samples were allowed to settle at RT for 20 min. Subsequently, the beads were washed twice with 70% *v*/*v* ethanol in water and once with acetonitrile. The beads were resuspended in 50 mM NH_4_HCO_3_ supplemented with trypsin (Mass Spectrometry Grade, Promega, Walldorf, Germany) at an enzyme-to-protein ratio of 1:25 *w*/*w* and incubated overnight at 37 °C. After overnight digestion, acetonitrile was added to the samples to reach a final concentration of 95% *v*/*v*, followed by incubation at RT for 20 min. To increase the yield, supernatants derived from this initial peptide-binding step were additionally subjected to the SP3 peptide purification procedure [69]. Each sample was washed with acetonitrile. To recover bound peptides, paramagnetic beads from the original sample and corresponding supernatants were pooled in 2% *v*/*v* dimethyl sulfoxide (DMSO) in water and sonicated for 1 min. After 2 min of centrifugation at 12,500 rpm and 4 °C, supernatants containing tryptic peptides were transferred into a glass vial for mass spectrometry and acidified with 0.1% *v*/*v* formic acid.

### 2.14. Liquid Chromatography-Mass Spectrometry (LC-MS) Analysis

Tryptic peptides were separated using an Ultimate 3000 RSLCnano LC system (Thermo Fisher Scientific, Waltham, MA, USA) equipped with a PEPMAP100 C18 5 µm, 0.3 × 5 mm trap (Thermo Fisher Scientific, Waltham, USA) and an HSS-T3 C18 1.8 μm, 75 μm × 250 mm analytical reversed-phase column (Waters Corporation, Milford, CT, USA). Mobile phase A was water containing 0.1% *v*/*v* formic acid and 3% *v*/*v* DMSO. Peptides were separated by running a gradient of 2–35% mobile phase B (0.1% *v*/*v* formic acid, 3% *v*/*v* DMSO in ACN) over 40 min at a flow rate of 300 nL/min. The total analysis time was 60 min, including wash and column re-equilibration steps. The column temperature was set to 55 °C. Mass spectrometric analysis of eluting peptides was conducted on an Orbitrap Exploris 480 (Thermo Fisher Scientific, Waltham, MA, USA) instrument platform. The spray voltage was set to 1.8 kV, the funnel RF level to 40, and the heated capillary temperature was at 275 °C. Data were acquired in the data-dependent acquisition (DDA) mode targeting the 10 most abundant peptides for fragmentation (Top10). Full MS resolution was set to 120,000 at *m*/*z* 200, and full MS automated gain control (AGC) target to 300% with a maximum injection time of 50 ms. Mass range was set to *m*/*z* 350–1500. For MS2 scans, the collection of isolated peptide precursors was limited by an ion target of 1 × 105 (AGC target value of 100%) and maximum injection times of 25 ms. Fragment ion spectra were acquired at a resolution of 15,000 at *m*/*z* 200. The intensity threshold was kept at 1E4. The isolation window width of the quadrupole was set to 1.6 *m*/*z*, and the normalized collision energy was fixed at 30%. All data were acquired in profile mode using positive polarity. Samples were analyzed in three technical replicates.

### 2.15. Data Analysis and Label-Free Quantification

DDA raw data acquired with the Exploris 480 were processed with MaxQuant (version 2.0.1) [70,71], using the standard settings and label-free quantification (LFQ) enabled for each parameter group, i.e., control and affinity-purified samples (LFQ min ratio count 2, stabilize large LFQ ratios disabled, match-between-runs). Data were searched against the forward and reverse sequences of the *P. falciparum* proteome (UniProtKB/TrEMBL, 5445 entries, UP000001450, release April 2020) and a list of common contaminants. For peptide identification, trypsin was set as protease allowing two missed cleavages. Carbamidomethylation was set as fixed and oxidation of methionine as well as acetylation of protein N-termini as variable modifications. Only peptides with a minimum length of 7 amino acids were considered. Peptide and protein false discovery rates (FDR) were set to 1%. In addition, proteins had to be identified by at least two peptides. Statistical analysis of the data was conducted using the Student’s *t*-test, which was corrected by the Benjamini–Hochberg (BH) method for multiple hypothesis testing (FDR of 0.01). In addition, proteins in the affinity-enriched samples had to be identified in all three biological replicates and to show at least a two-fold enrichment as compared to the controls. The datasets of protein hits were further edited by verification of the gene IDs and gene names via the PlasmoDB database (www.plasmodb.org; [55]; accessed on 1 May 2022). PlasmoDB gene IDs were extracted from the fasta headers provided by mass spectrometry using R software version 4.2.0 and verified manually. Proteins containing signal peptides and transmembrane domains were predicted by the “signalHsmm R” package and the TMHMM-2.0 software, respectively, and eventually removed from the considered list of potential interaction partners. To finally obtain a more curated list of proteins that may potentially be the interactors of *Pf*SAMS, the gene IDs of those proteins were subjected to Gene Ontology (GO) terms analysis for cellular components. At a *p*-value of 0.01, proteins predicted to localize in the nucleus and cytoplasm based only on curated information in PlasmoDB were considered for further analysis. In addition, a Kyoto Encyclopedia of Genes and Genomes (KEGG) search was carried out by uploading the 1114 interactors in the KEGG database (https://www.genome.jp/kegg/kegg1b.html; accessed on 20 June 2022), using Cluster Profiler version 4.0. A search against *P. falciparum*-specific pathways was plotted. Further, a network analysis was conducted using the STRING database (version 11.0) [72], using default settings and confidence of 0.009. The analysis of log2 ratio values of the identified *Pf*SAMS interactors was also conducted using R software version 4.2.0. Text mining was carried out using R packages “tidytext”, stringr” and “tidyr”, while plots were generated with the R package “ggplot2”.

### 2.16. Data Availability

The mass spectrometry proteomics data have been deposited to the ProteomeXchange Consortium (http://proteomecentral.proteomexchange.org; accessed on 13 July 2022) via the jPOST partner repository [73] with the dataset identifiers PXD034111 (ProteomeXchange) and JPST001602 (jPOST).

### 2.17. Statistical Analysis

Data are presented as mean ± SD. Statistical differences were determined using one-way ANOVA with post hoc Bonferroni multiple comparison test or unpaired tow-tailed Student’s *t*-test, as indicated. *p*-values < 0.05 were considered statistically significant. Significances were calculated using GraphPad Prism 5 and are represented in the figures as follows: ns, not significant *p* > 0.05; * *p* < 0.05; ** *p* < 0.01; *** *p* < 0.001.

## 3. Results

### 3.1. PfSAMS Is Expressed in the Asexual and Sexual Blood Stages of P. falciparum

In *P. falciparum*, SAMS is a 45-kDa protein (PF3D7_0922200; 402 aa) encoded by a single gene on chromosome 9. For functional characterization, we generated a conditional *Pf*SAMS-HA-KD line (using the pSLI-HA-*glmS* vector) by fusing the sequences coding for a hemagglutinin A (HA)-tag and for the *glmS* element to the 3′-coding region of *pfsams* (Figure S1A). In the *Pf*SAMS-HA-KD, the *pfsams* mRNA is expressed under the control of the *glmS* ribozyme [74], which catalyzes its own cleavage in the presence of GlcN and thus triggers transcript degradation. Vector integration was confirmed by diagnostic PCR (Figure S1B). Immunoblotting with an anti-HA antibody confirmed the synthesis of an HA-tagged *Pf*SAMS fusion protein in the untreated *Pf*SAMS-HA-KD line, running at a molecular weight of ~ 50 kDa, while no signal was detected in WT NF54 (Figure S1C).

**Figure 1:**
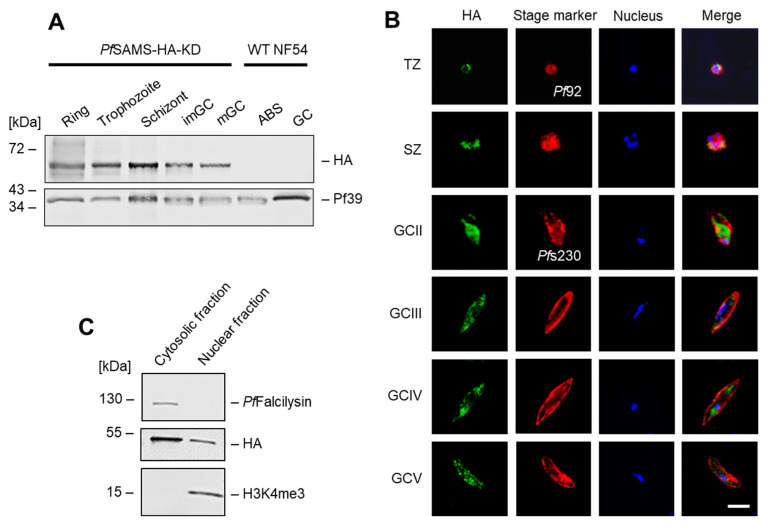
Expression and localization of *Pf*SAMS-HA in the *P. falciparum* blood stages. (**A**) Expression of *Pf*SAMS-HA in blood-stage parasites. Lysates of asexual blood stages (ABS) of the ring, trophozoite, and schizont stage and of immature (imGC) and mature (mGC) gametocytes of the *Pf*SAMS-HA-KD parasite line were immunoblotted with rat anti-HA antibody to detect *Pf*SAMS-HA (~50 kDa); immunoblotting with antisera directed against *Pf*39 (39 kDa) was used as loading control. (**B**) Localization of *Pf*SAMS-HA in the asexual blood stages and gametocytes. Immunofluorescence assays were employed, using rat anti-HA antibody, to detect *Pf*SAMS-HA in trophozoites (TZ), schizonts (SZ), and gametocytes (GC) of stages II–V (green), of the *Pf*SAMS-HA-KD parasite line. Counterlabeling of the asexual blood stages was done using mouse anti-*Pf*92 antisera and of gametocytes with rabbit anti-*Pf*s230 antisera (red). The nuclei were labeled with Hoechst 33342 nuclear stain (blue). Bar; 5 µm. (**C**) Subcellular fractionation of *Pf*SAMS-HA parasites. Nuclear and cytosolic fractions were obtained from schizonts of the *Pf*SAMS-HA-KD parasite line and the fractions were immunoblotted with rat anti-HA antibody. Purity of the nuclear and cytosolic fractions was confirmed by immunoblotting with mouse antiserum directed against the cytosolic protease *Pf*Falcilysin (138 kDa) and rabbit antibody directed against the nuclear histone H3 mark H3K4me3 (15 kDa), respectively. The results (**A**–**C**) are each representative of three independent experiments.

The untreated *Pf*SAMS-HA-KD line was used for in-depth expression analysis. Western blotting, using an anti-HA antibody, demonstrated a prominent expression of *Pf*SAMS-HA in the asexual blood stages as well as in the immature and mature gametocytes, while no protein band was detected in the WT NF54 asexual blood stages and gametocytes (Figure 1A). Immunofluorescence assays further showed that *Pf*SAMS localizes to both nucleus and cytoplasm in these stages (Figure 1B), while WT NF54 blood-stage parasites did not show any labeling (Figure S2). Subcellular fractioning confirmed that *Pf*SAMS-HA localizes to the cytosolic and nuclear fractions of the blood-stage parasite (Figure 1C). The purity of the fractions was confirmed by immunoblotting with antibodies against the cytosolic protease *Pf*Falcilysin [58] and the nuclear histone H3 (as identified by the methylation mark H3K4me3).

### 3.2. PfSAMS Deficiency Impairs Intraerythrocytic Growth and Gametocyte Development

**Figure 2:**
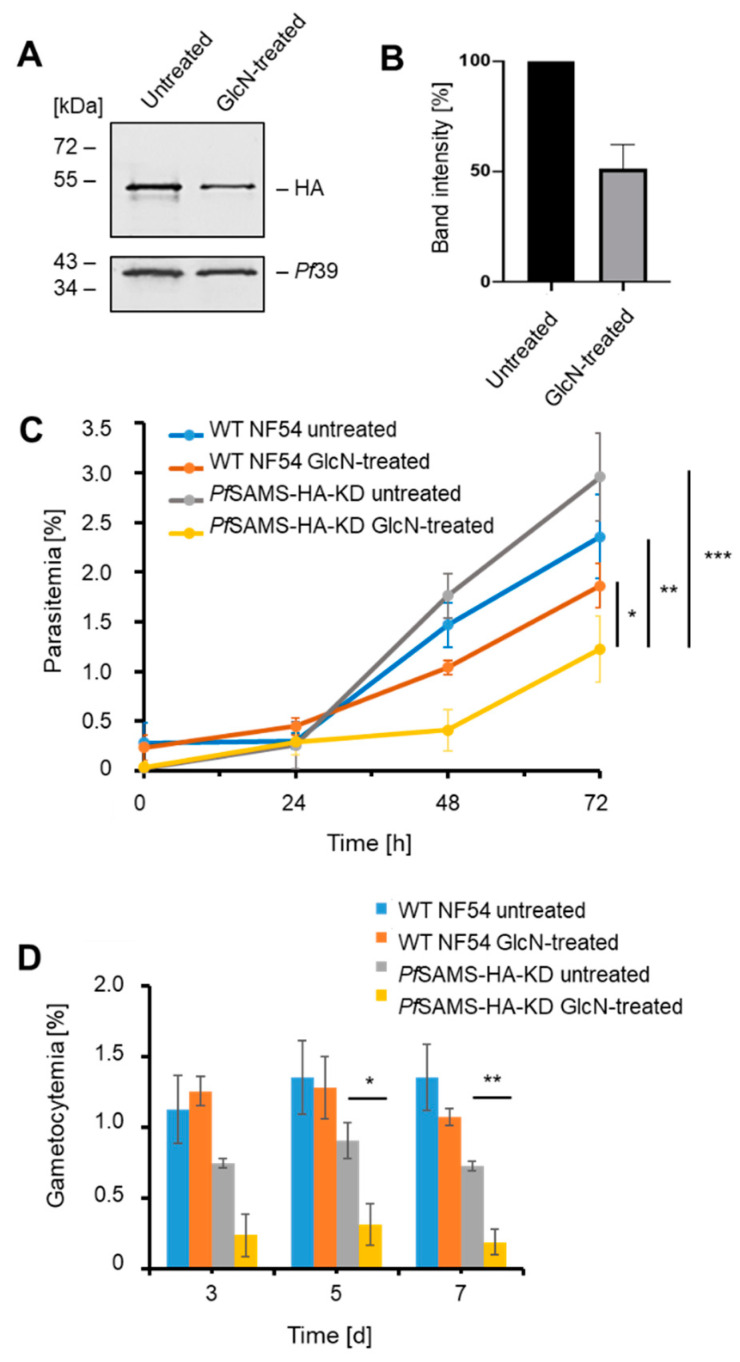
The effect of *Pf*SAMS-HA deficiency on intraerythrocytic growth and gametocyte development. (**A**) Downregulation of *Pf*SAMS-HA levels. Schizont cultures of the *Pf*SAMS-HA-KD parasite line were treated with 5 mM GlcN for 72 h to determine knockdown of *PfS*AMS. Lysates were immunoblotted with rat anti-HA antibody to detect *Pf*SAMS-HA (~50 kDa); immunoblotting with rabbit anti-*Pf*39 antisera (39 kDa) was used as loading control. (**B**) Quantification of *Pf*SAMS-HA levels following knockdown. *Pf*SAMS-HA levels were evaluated between the GlcN-treated and untreated *Pf*SAMS-HA-KD parasites in three independent Western blots based on the band intensity as estimated with ImageJ software. *Pf*39 levels were used for normalization (set to 100%). (**C**) Asexual blood-stage replication following *Pf*SAMS-HA knockdown. A highly synchronized *Pf*SAMS-HA-KD parasites ring stage culture was set up with a parasitemia of 0.25% and treated with 5 mM GlcN. The parasitemia was determined by Giemsa smears at 0 h, 24 h, 48 h, and 72 h. Untreated *Pf*SAMS-HA-KD cultures as well as GlcN-treated and untreated WT NF54 cultures were used as controls. (**D**) Gametocyte development following *Pf*SAMS-HA knockdown. A synchronized parasite culture of the *Pf*SAMS-HA-KD line was treated with 5 mM GlcN for 72 h, and gametocytogenesis was subsequently induced with lysed blood cells while the GlcN treatment was continued. The gametocytemia was evaluated by Giemsa smears on days 3, 5, and 7. Untreated *Pf*SAMS-HA-KD cultures as well as GlcN-treated and untreated WT NF54 cultures were used as controls. Statistical analyses were performed using one-way ANOVA; *p* > 0.05; * *p* < 0.05; ** *p* < 0.01; *** *p* < 0.001. The results are representative of three (**C**) and one (**D**) independent experiments.

To induce the conditional knockdown of *Pf*SAMS-HA synthesis, asexual blood-stage parasites were treated with 5 mM GlcN for 72 h. Upon GlcN addition, *Pf*SAMS-HA levels were significantly reduced to 50.1 ± 7.27% compared to the untreated control, as shown by quantitative Western blotting (Figure 2A,B).

The *Pf*SAMS-HA-KD line was then investigated for intraerythrocytic development of the asexual blood stages. Growth assays revealed that the parasitemia of synchronized asexual blood stages treated with 5 mM GlcN was significantly reduced after 72 h when compared to untreated *Pf*SAMS-HA-KD parasites and GlcN-treated or untreated WT NF54 parasites (Figure 2C). No significant difference in the parasitemia was observed when GlcN-treated and untreated WT NF54 were compared at 72 h. In addition, quantification of ring, trophozoite, and schizont stages at different time points did not reveal a major effect of GlcN-treatment on parasite stage development of *Pf*SAMS-HA-KD and WT NF54 parasites (Figure S3), suggesting that the lack of *Pf*SAMS affects parasite viability, but does not delay the blood-stage cycle.

*Pf*SAMS-HA downregulation further affected gametocyte development. When *Pf*SAMS-HA-KD gametocytes were treated with GlcN prior to gametocyte commitment and during gametocytogenesis, the numbers of gametocytes drastically reduced over a period of seven days compared to untreated *Pf*SAMS-HA-KD gametocytes (Figure 2D), while no significant difference in gametocyte numbers was observed between GlcN-treated and untreated WT NF54 parasites.

**Figure 3:**
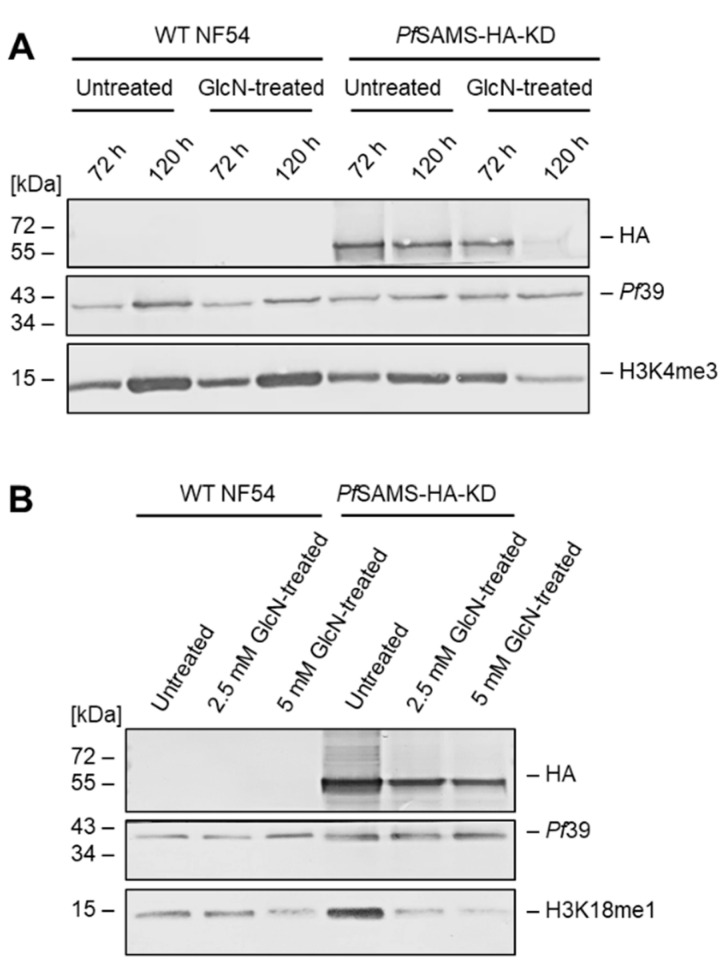
Effect of *Pf*SAMS deficiency on histone H3 methylation in the asexual blood stages. Lysates of untreated and GlcN-treated asexual blood-stage cultures in (**A**), 72 h and 120 h, using 5 mM GlcN; in (**B**), 72 h, using 2.5 or 5 mM GlcN) of the *Pf*SAMS-HA-KD line and WT NF54 were immunoblotted with rat anti-HA antibodies to detect *Pf*SAMS-HA (~50 kDa). Histone H3 methylation was detected by immunoblotting with rabbit antibodies directed against H3K4me3 (15 kDa) (**A**) and H3K18me1 (15 kDa) (**B**). Rabbit antisera directed against *Pf*39 (39 kDa) was used as loading and viability control. The results (**A**,**B**) are each representative of two independent experiments.

Because *Pf*SAMS has been assigned a major role in histone methylation during transcriptional regulation, we investigated the effect of *Pf*SAMS deficiency on two histone H3 methylation marks, for which efficient antibodies are available, i.e., H3K4me3 and H3K18me1. Asexual blood-stage parasite cultures were treated with GlcN for 72 or 120 h at concentrations of 2.5 mM or 5 mM. Lysates were immunoblotted with anti-HA antibodies to detect *Pf*SAMS-HA in the samples, while the H3K4me3 and H3K18me1 methylation marks were detected with the respective antibodies (Figure 3A,B). Treatment of the *Pf*SAMS-HA-KD line with GlcN affected the *Pf*SAMS-HA levels, as expected, and further reduced the methylation status for the two methylation marks. The levels of *Pf*39, used as a loading and viability control, were not affected by the GlcN treatment. Further, no down-regulation in the histone methylation marks due to GlcN-treatment was observed in WT NF54 parasites.

### 3.3. BirA-Tagging of PfSAMS Results in Protein Biotinylation in Blood Stage Parasites

For further characterization of *Pf*SAMS, we generated transfectant lines episomally expressing a *Pf*SAMS-GFP-BirA fusion protein. Blood stage parasites were transfected with the vectors pARL-*Pf*SAMS-*pfama1*-GFP-BirA or pARL-*Pf*SAMS-*pffnpa*-GFP-BirA, whereby the expression of *Pf*SAMS-GFP-BirA was either controlled by the asexual blood stage-specific *pfama1* or the gametocyte-specific *pffnpa* promotor (Figure S4A,B). The presence of the vector in the respective transfectant was confirmed by diagnostic PCR (Figure S4C,D).

**Figure 4:**
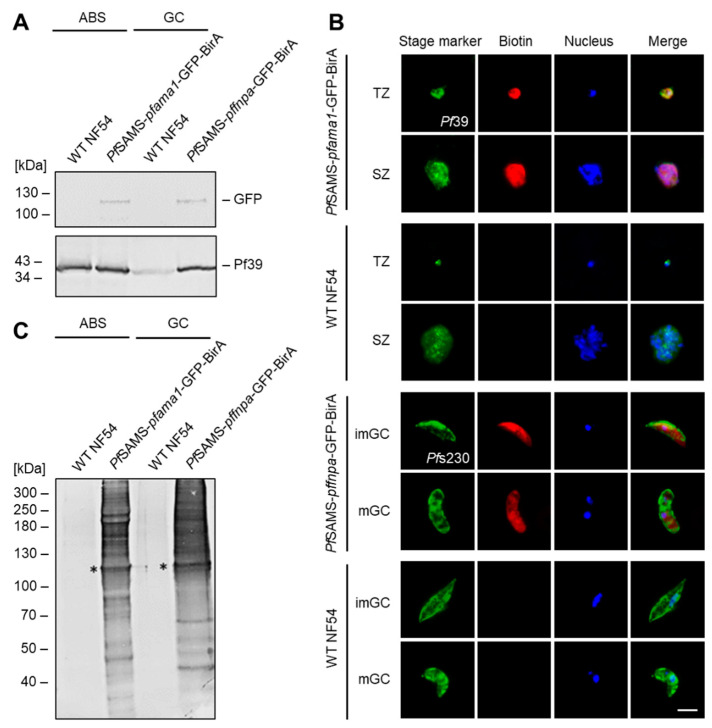
Generation and verification of *Pf*SAMS-GFP-BirA parasite lines. (**A**) Verification of *Pf*SAMS expression. Lysates of asexual blood stages (ABS) of the *Pf*SAMS-*pfama1*-GFP-BirA and gametocytes (GC) of the *Pf*SAMS-*pffnpa*-GFP BirA lines were subjected to Western blotting to detect *Pf*SAMS-GFP-BirA (108 kDa), using mouse anti-GFP antibody. Immunoblotting with rabbit antisera directed against *Pf*39 (39 kDa) served as a loading control. WT NF54 lysates were used as negative controls. (**B**) Localization of biotinylated proteins in the *Pf*SAMS-GFP-BirA blood stages. Asexual blood stages of the *Pf*SAMS-*pfama1*-GFP-BirA line and gametocytes of the *Pf*SAMS-*pffnpa*-GFP-BirA line were treated with 50 μM biotin for 24 h. WT NF54 was used as a negative control. Immunofluorescence assays were employed, using mouse antibodies directed against *Pf*39 to highlight trophozoites (TZ), and schizonts (SZ) and rabbit antisera directed against *Pf*s230 to highlight immature (imGC) and mature (mGC) gametocytes (green). Biotinylated proteins were immunolabeled using fluorophore-conjugated streptavidin (red). The nuclei were labeled with Hoechst 33342 nuclear stain (blue). Bar; 5 µm. (**C**) Detection of biotinylated proteins in the *Pf*SAMS-GFP-BirA lines. Lysates of asexual blood stages (ABS) from the *Pf*SAMS-*pfama1*-GFP-BirA line and gametocytes (GC) of the *Pf*SAMS-*pffnpa*-GFP-BirA line were produced following treatment of the cultures with 50 μM biotin for 24 h. Streptavidin coupled to alkaline phosphatase was used for immunoblotting. Asterisks indicate the expected band for *Pf*SAMS-GFP-BirA (~108 kDa). The results are representative of two (**A**,**C**) or three (**B**) independent experiments.

Western blot analysis demonstrated the presence of a *Pf*SAMS-GFP-BirA fusion protein in lysates of asexual blood stages and gametocytes of the respective transfectant lines (i.e., *Pf*SAMS-*pfama1*-GFP-BirA or *Pf*SAMS-*pffnpa*-GFP-BirA), running at the expected molecular weight of ~108 kDa (Figure 4A), while no such signal was detected in WT NF54 parasites. Similarly, immunofluorescence analyses demonstrated the expression of the *Pf*SAMS-GFP-BirA fusion protein by labeling with anti-GFP antibodies and confirmed the presence of tagged *Pf*SAMS in the cytoplasm and nuclear region of asexual blood-stage parasites and gametocytes (Figure S5A). In WT NF54 parasites, no GFP labeling was detected.

Subsequent immunolabeling experiments using fluorophore-conjugated streptavidin unveiled the presence of biotinylated proteins in the cytoplasm and nuclear regions of asexual blood-stage parasites and gametocytes of the respective transfectant lines, when these were incubated with 50 μM biotin for 24 h prior to fixation (Figure 4B); the WT NF54 control was also incubated with 50 μM biotin for 24 h prior to fixation and no biotinylated proteins were detected. Noteworthy, the signals for immunolabeled *Pf*SAMS-GFP-BirA and immunolabeled biotinylated proteins overlapped (Figure S5B), confirming that proteins in the proximity of *Pf*SAMS-GFP-BirA were biotinylated. When lysates of the biotin-treated cultures were subjected to Western blot analysis, using streptavidin conjugated to alkaline phosphatase, multiple protein bands indicative of biotinylated proteins were detected, including a protein band at ~108 kDa, representing biotinylated *Pf*SAMS-GFP-BirA (Figure 4C). No biotin–positive protein bands were detected in WT NF54 samples.

### 3.4. The PfSAMS Interactome Comprises Nuclear and Cytosolic Proteins Involved in Vital Cellular Functions

**Figure 5:**
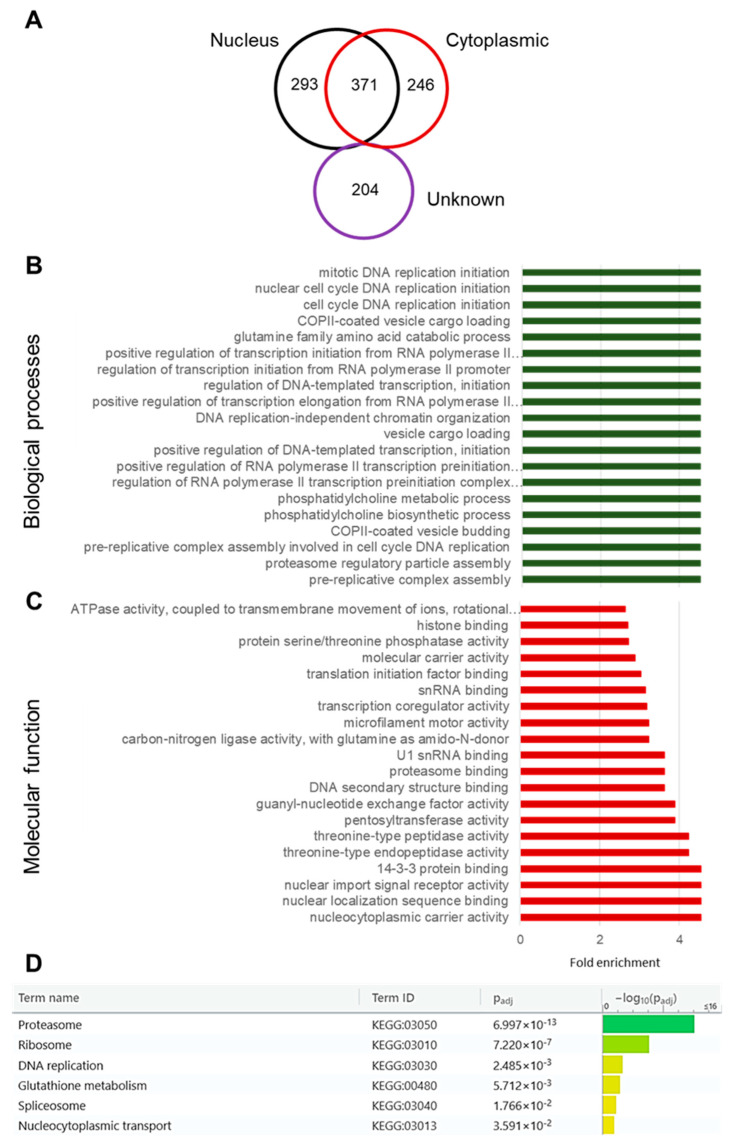
Functional prediction analysis of putative interaction partners of *Pf*SAMS. A total of 1114 identified potential interactors of *Pf*SAMS were matched to the PlasmoDB database entries for their cellular localization, biological processes, and molecular function based on GO term enrichment analyses of both computed and curated data at a *p*-value of 0.01. (**A**) Venn diagram showing the predicted nuclear and cytoplasmic localization of the identified interactors, including proteins of unknown localization. The GO categories “Nucleus” (GO:0005634) and “Cytoplasm” (GO:0005737) were considered. (**B**,**C**) Interactors of the 20 highest scoring biological processes (**B**) and 20 highest scoring molecular functions (**C**) as predicted by GO enrichment analysis, sorted by fold enrichment. (**D**) KEGG analysis of *Pf*SAMS interactors.

To determine the potential interaction network of *Pf*SAMS, the *Pf*SAMS-*pfama1*-GFP-BirA line was then employed in BioID analysis. The fusion of *Pf*SAMS with the *E. coli* biotin ligase BirA enables the covalent biotinylation of proximate proteins in situ, which can then be detected and affinity-purified using streptavidin [75]. To analyze the *Pf*SAMS interactome by BioID, the biotinylated proteins were purified from schizont lysates of the *Pf*SAMS-*pfama1*-GFP-BirA transfectant line via streptavidin-coated beads. Pull-down samples from WT NF54 lysates were used as a control. The biotinylated proteins were then prepared for mass spectrometry in triplicate. At least 1114 proteins were identified to be significantly biotinylated in the *Pf*SAMS-GFP-BirA samples (Table S2). Based on prediction methods, proteins with signal peptides and those putatively localizing in the membranes were removed, and only proteins that match the curated cellular localization as indicated by PlasmoDB entries were considered. Out of the 1114 potential interactors of *Pf*SAMS, 293 could be assigned to the nucleus, 246 are cytoplasmic, and 371 proteins were reported for both nucleus and cytoplasm (Figure 5A; Table S3). For a further 204 proteins, the subcellular localization could not be predicted.

GO terms analysis was carried out to depict the functions of the identified proteins. The identified GO terms confirmed the subcellular localization of *Pf*SAMS in both nucleus and cytoplasm and highlighted that *Pf*SAMS interactors are involved in vital biological processes such as cell cycle control, DNA replication, and transcription, but also in PC biosynthesis, vesicle trafficking, and proteasome assembly (Figure 5B; Table S3). Molecular functions predicted for the *Pf*SAMS interactors include ATPase activity, histone binding, transcriptional regulation, and translation initiation, but also proteasome binding, nuclear trafficking, and protease/peptidase activities (Figure 5C; Table S3). Furthermore, a KEGG analysis assigned the *Pf*SAMS interactors to the proteasome, ribosome, and spliceosome as well as to DNA replication, glutathione metabolism, and nucleocytoplasmic transport (Figure 5D). In total, the functional prediction analyses suggest the roles of *Pf*SAMS in vital processes in both the nuclear and cytosolic compartments such as DNA replication, transcriptional regulation, chromatin remodeling, and translation as well as proteostasis.

**Figure 6:**
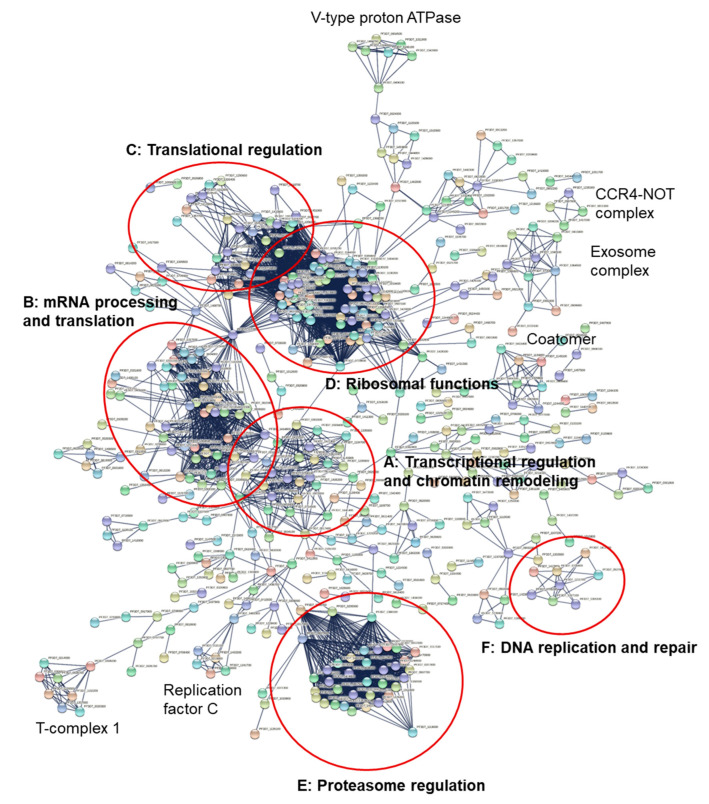
Network analysis of the putative interactors of *Pf*SAMS. A list of 1114 query proteins was evaluated for potential interactions using the String database. A network of potential *Pf*SAMS interactors was predicted with a confidence level of 0.009. A Markov Clustering (MCL) algorithm was used to describe possible clusters with an inflation parameter of 3. Based on the physical interaction among only the query proteins, different clusters were identified built on the first shell of interaction with the following functions: (**A**) transcriptional regulation and chromatin remodeling with at least 20 member proteins; (**B**) mRNA processing and translation, including proteins of the spliceosome comprised of a subnetwork of more than 29 proteins, in contact with cluster **A** (**C**) Translational regulation with a cluster of translation regulation factors, that include mainly eukaryotic translation initiation factors; (**D**) ribosomal functions with ribosomal proteins presenting the largest cluster of the network, in contact with cluster **C** (**E**) proteasome regulation with a cluster of proteasome subunits with at least 38 connected proteins; (**F**) DNA replication and repair in a subnetwork of mainly mini-chromosome maintenance (MCM) proteins and DNA helicases, with no clear link to the other clusters. Smaller clusters of distinct protein complexes are indicated. Detailed information about individual proteins clustering in each subnetwork is provided in Table S4.

A Markov Clustering algorithm was applied to the 1114 potential interactors of *Pf*SAMS with a confidence level of 0.009. Based on the physical interaction among only the query proteins, different clusters were identified, including six main clusters (Figure 6A–F, Table S4) with functions in transcriptional regulation and chromatin remodeling (A), mRNA processing and translation (B), translational regulation (C), ribosomal functions (D), proteasome regulation (E), and DNA replication and repair (F) (see also: https://string-db.org/cgi/network?taskId=bh6wkc1a34Ct&sessionId=bLg1dsxSNHTf; network generated on 1 May 2022). Smaller clusters define distinct protein complexes, such as the CCR4-NOT complex, the exosome complex, or the T-complex 1 (Figure 6).

Transcriptional regulation and chromatin remodeling factors were identified with at least 20 interacting proteins (Figure 6 and Figure S6). Among the key interactors, the putative protein arginine N-methyltransferase 5 (PRMT5) [PF3D7_1361000] that belongs to the class I-like SAM-binding methyltransferase superfamily shows interaction with known chromatin remodeling regulators such as histone deacetylase HDAC1 [PF3D7_0925700] and spliceosome proteins such as the putative small nuclear ribonucleoprotein-associated protein B [PF3D7_1414800] and small nuclear ribonucleoprotein Sm D1 important for the pre-mRNA metabolism [PF3D7_1125500]. The network further demonstrates the interaction of transcription factors with histones as mediated by putative FACT complex subunit SPT16 [PF3D7_0517400] that interacts with histone H2B [PF3D7_1105100], histone H4 [PF3D7_1105000], and histone transferases such as the putative histone-lysine N-methyltransferase 1 [PF3D7_0629700] and acetyltransferases such as histone acetyltransferase GCN5 [PF3D7_0823300] and the putative histone acetyltransferase [PF3D7_0416400]. GCN5 also shows a predicted physical interaction with 26S proteasome proteins, DNA helicases, and the putative DNA methyltransferase 1-associated protein 1 [PF3D7_0628600], suggestive of a model of interactions, where histone modification enzymes, the ubiquitin-proteasome system (UPS) and *Pf*SAMS closely act together to regulate transcription and proteostasis.

The cluster of transcription regulation factors and the spliceosome proteins are linked by the putative pre-mRNA-processing protein 45 [PF3D7_0218700], DNA-directed RNA polymerase subunit beta [PF3D7_0215700], putative small nuclear ribonucleoprotein-associated protein B [PF3D7_1414800] and probable DNA-directed RNA polymerase II subunit RPB11 [PF3D7_1304900]. Proteins of the spliceosome comprise a subnetwork of at least 29 proteins (Figure 6 and Figure S6; Table S4). The ribonucleoprotein-associated protein SNU13 [PF3D7_1123900], a common component of the spliceosome and rRNA processing machinery that belongs to the eukaryotic ribosomal protein eL8 family, represents the intermediary between interactions of the spliceosome protein and translation initiation factor cluster, together with the eukaryotic initiation factor 4A that belongs to the DEAD box helicase family. A cluster of translation regulation factors that include mainly eukaryotic translation initiation factors is shown in tight interaction with ribosomal proteins comprised of at least 36 connected proteins. The cluster of proteasome-associated proteins with at least 38 connected proteins shows physical interactions with the putative DNA repair protein RAD23 [PF3D7_1011700], a node that plays an intermediate link between 26S proteasome proteins and transcription regulation factors with DNA polymerases. RAD23, as a nuclear protein, shows a potential physical interaction with the proteasome, transcription factors, cell division cycle proteins such as the putative cell division cycle protein 48 homolog [PF3D7_0619400] and the E3 ligases important in the UPS system, which may also hypothesize a possible interaction of *Pf*SAMS, chromatin remodeling proteins, and the UPS system to regulate gene expression and cell growth (Figure 6 and Figure S6, Table S4).

A small subnetwork of proteins that are putatively involved in DNA replication and repair has been identified. At least eight proteins of the mini-chromosome maintenance complex-binding proteins (MCM) and helicases show interconnection in this subnetwork with no clear link to the other clusters (Figure 6; Table S4). Origin recognition complex subunit 2 protein (*Pf*Orc2) [PF3D7_0705300] found in this subnetwork is a DNA replication initiation protein that can complement yeast Orc2. *Pf*Orc2 is known to be processed in the endoplasmic reticulum and trafficked to the nucleus through the classical secretory pathway in *P. falciparum*. The nuclear import of *Pf*Orc2C depends on importin-α/β, also identified in the *Pf*SAMS interactors [76]. Other members of this subnetwork include DNA replication licensing factors MCM3, MCM4, MCM5, and MCM7 of the MCM family.

The identified interaction partners show a prediction of interaction in a large network and subnetworks only among themselves without the intermediary of other proteins that do not belong to the query list of identified *Pf*SAMS interactors, based on the data mined in the String database. A PPI enrichment *p*-value of 3 × 10^9^ was computed with a higher number of edges (4024) in the interactors than expected (3666) on random distribution and average local clustering coefficient of 0.327, indicating that the identified proteins have more interactions among themselves than would be expected for a random set of proteins of the same group size and degree distribution drawn from the *P. falciparum* 3D7 genome.

Selected interactors were further grouped by biological functions (Table S5). A high number of interactors belong to the methyltransferases; these include the genes coding for five of the ten SET (Su(var)3-9-’Enhancer of zeste-Trithorax)-domain-containing lysine-specific histone methyltransferases [77,78], i.e., *Pf*SET1, *Pf*SET3, *Pf*SET6, *Pf*SET7, and *Pf*SET9, as well as the three protein arginine methyltransferases *Pf*PRMT1, *Pf*PRMT5 and *Pf*CARM1 [79]. In addition, we identified the interaction of *Pf*SAMS with various DNA-directed polymerase subunits, transcription elongation factors, and translation elongation factors as well as with the enzyme *Pf*PMT that is important for alternative PC synthesis (Table S5). Importantly, we were also able to identify potential interactions with components of the polyamine biosynthesis pathway, such as the spermidine synthase SpdSyn and the S-adenosylmethionine decarboxylase/ornithine decarboxylase AdoMetDC/ODC (Table S5). In addition, multiple subunit components of the plasmodial proteasome were identified as interactors of *Pf*SAMS (Table S5). Together with the assigned functional predictions in proteasome, protease, and peptidase activities (Figure 5C,D), an important role of *Pf*SAMS in proteostasis can be anticipated. Our data further show a potential interaction of *Pf*SAMS with at least nine of the 27 members of the AP2 DNA-binding protein family of *P. falciparum* with high log2 ratio values as compared to other interactors, including AP2-G, the key regulator of sexual commitment (Table S5). Because we identified interactions of *Pf*SAMS with the sexual commitment regulator AP2-G as well as with *Pf*PMT (see above), which is important for PC synthesis during sexual commitment, the combined data suggest a crucial role of *Pf*SAMS during the initiation of gametocytogenesis.

Lastly, we summarized the interactome data based on the peptide count and the log2 ratio of the identified proteins. The *Pf*SAMS interactors could be grouped into two distinct categories based on the log2 ratio. The first group includes proteins with a log2 ratio of less than 10, while the other group includes proteins exhibiting a log2 ratio of greater than 25 (Figure S7A). Although the comparison of the log2 ratio among subcellular compartments could not show a clear difference (Figure S7B), the group of proteins with a log2 ratio higher than 25 particularly included proteins with functions in the nucleus such as transcriptional regulation and mRNA processing (Figure S7; compare Table S5), suggesting a particular involvement of *Pf*SAMS in nuclear processes. A further analysis of the two categories of *Pf*SAMS interactors was conducted based on the classes of enzymes that potentially interact with *Pf*SAMS. Using a text-mining algorithm, different classes of interacting enzymes were identified and plotted based on their absolute count and further compared based on their log2 ratio (Figure S8A,B). Classes of enzymes such as kinases, polymerases, methyltransferases, GTPases, helicases, and phosphatases were significantly grouped in the category of log2 ratio higher than 25 as compared to other enzyme classes, supporting the important role of *Pf*SAMS in gene expression and the post-translational modification of proteins.

## 4. Discussion

The sulfonium compound SAM participates in a variety of biochemical processes with vital functions for the eukaryotic cell. It is the main methyl donor reagent for significant methylation reactions that are crucial for the epigenetic regulation of gene expression and for further metabolic pathways such as polyamine synthesis. SAM is synthesized through the reaction of methionine with ATP, catalyzed by SAMS, and is an important metabolite that, due to its dependency on methionine availability, can act as a nutrition and stress sensor in the cell [3].

In the malaria parasite *P. falciparum*, the SAM-synthesizing enzyme *Pf*SAMS is encoded by a single gene, making the parasite particularly vulnerable to methionine and SAM availability. Although this genetic bottleneck emphasizes the crucial role of *Pf*SAMS for parasite viability, the enzyme is not yet well investigated in malaria parasites. Therefore, we aimed to study the role of *Pf*SAMS in the parasite blood stages and to unveil the *Pf*SAMS interactome.

Using two independently generated *Pf*SAMS-tagging transfectant lines, we show that *Pf*SAMS is present in all blood stages of *P. falciparum* and is localized in the cytoplasm and nucleus of the parasite, pointing to its diverse functions. These expression data are in accord with data from other eukaryotes, where SAMS or MAT enzymes have also been reported to localize to the nucleus and cytoplasm of the cell [80]. To gain information on the role of *Pf*SAMS in the *P. falciparum* blood stages, we generated a pSLI-*glmS*-based *Pf*SAMS-HA-KD line. The induced deficiency of *Pf*SAMS reduced the methylation of two known methylation marks, H3K4me3 and H3K18me1 [81]. Particularly H3K4me3 has previously been studied extensively since it is linked to the active state of *var* genes [12,21]. Our histone methylation data demonstrate the methylation activity of *Pf*SAMS in the parasite blood stages and confirm its role in histone H3 methylation modifications. We further show that *Pf*SAMS deficiency impairs intraerythrocytic growth as well as gametocyte development, highlighting the vital role of the enzyme for both the asexual blood stages and gametocytes. Noteworthy, another recently generated *glmS*-based *Pf*SAMS-KD was investigated for its effect on gametocyte commitment and showed that *Pf*SAMS deficiency and hence lack of SAM forces *P. falciparum* into the sexual pathway [45]. The effect of *Pf*SAMS deficiency on gametocyte maturation, however, has not been investigated in this study.

To understand the protein interaction network involving *Pf*SAMS, we generated a transfectant line expressing a *Pf*SAMS-GFP-BirA-fusion protein to be used for BioID analysis. The transfectant line was treated with biotin at the ring stage, and after 24 h, biotinylated proteins were purified, using streptavidin beads, and detected by mass spectrometry. Following the exclusion of proteins with a signal peptide and transmembrane proteins, which would be expected to follow the secretory pathway and hence not be available to *Pf*SAMS, a total of 1114 hits were identified. The large number of potential interactors indicates the diverse involvement of *Pf*SAMS in many processes. Most of the hits were proteins assigned to the nucleus and the cytoplasm in accordance with the subcellular localization of *Pf*SAMS in the blood stages.

GO, KEGG, and String network analyses as well as functional classification strategies assigned *Pf*SAMS to six major functions, i.e., (1) DNA replication and repair; (2) transcriptional regulation and chromatin modeling; (3) translational regulation and protein synthesis; (4) protein processing and proteasome regulation; (5) PC metabolism; and (6) polyamine synthesis. Noteworthy, the clusters themselves are arranged in various sub-clusters, and the presence of multiple clusters and sub-clusters within the interactome suggests that *Pf*SAMS synthesizes SAM in locally confined subcellular compartments or microbodies rather than producing SAM that would be freely trafficked to other compartments as previously thought.

The link of *Pf*SAMS to epigenetic control was to be expected since methylation of histones, particularly H3, as well as of DNA and RNA has previously been reported for the asexual and sexual blood-stage parasites [12,20,21,25,46,48,49,81,82,83]. In accord with these findings, the interaction of *Pf*SAMS with five of the ten known *Pf*SET proteins, i.e., *Pf*SET1, *Pf*SET3, *Pf*SET6, *Pf*SET7, and *Pf*SET9, as well as with ribosomal RNA methyltransferases and DNA methyltransferase-associated proteins was confirmed. In addition, an interaction between *Pf*SAMS and AP-2G, the transcriptional regulator of gametocyte commitment, was detected. In non-committed parasites, *ap2-g* expression is repressed by H3K9me3 methylation and HP1 binding, while during sexual commitment, the *ap2-g* locus is activated by HP1 release, a process promoted by GDV-1 [26,38,39].

Noteworthy, *Pf*SAMS appears to interact with multiple ApiAP2 proteins in addition to AP2-G, including AP2-I, AP2-L, AP-O5, AP2-EXP, and the AP2/ERF domain-containing protein. AP2-I has been linked to binding to genes coding for proteasome complex subunits [84]. On the other hand, the AP2/ERF domain-containing protein has been previously shown to be involved in DNA replication and gene regulation in the asexual blood stages. Additionally, AP2-EXP was previously suggested to bind to the 5′ upstream regions of *var* genes and hence to play a role in the virulence of the blood-stage parasites. RNA-Seq analysis in AP2-EXP mutant parasites revealed transcriptional changes in a subset of exported proteins encoded by clonally variant gene families characterized with an upregulation of RIFINs and STEVORs at the protein levels, highlighting the importance of the non-DNA-binding AP2 domain in functional gene regulation [85]. Using the rodent malaria parasite *P. berghei*, AP2-L was further demonstrated to be important for the development of liver-stage parasites [86], while the ortholog of AP2-O5 in *P. yoelii*, Pyap2-o5, plays a role in ookinete motility and early oocyst development [87].

The biotinylation of histone methyltransferases and transcription factors suggests their close location to *Pf*SAMS in the parasite nucleus. Such direct interaction of SAMS enzymes with DNA-binding proteins has been suggested to occur in higher eukaryotes as well. For example, human SAMS serves as a transcriptional corepressor by directly binding DNA-binding proteins such as MafK and Bach1, which recognize the Maf recognition element (MARE) upstream of the nucleosome, thus recruiting methyltransferases and locally producing SAM, which is needed for histone methylation at the downstream nucleosome [88,89].

While the interaction of *Pf*SAMS with AP-2G suggests its involvement in gametocyte commitment, this event is particularly triggered by the lack of lysoPC in the medium. In fact, lysoPC deficiency forces the blood-stage parasites to synthesize PC by triple-methylation of phospho-ethanolamine using ethanolamine [30,43], and this pathway involves the enzyme *Pf*PMT, another interaction partner of *Pf*SAMS. In accord with these findings, lack of lysoPC in the medium results in increased *Pf*PMT expression and decreased SAM levels, while *Pf*PMT deficiencies increase intracellular SAM levels and repress sexual commitment, as has been described recently [45].

The network analysis further reveals the involvement of *Pf*SAMS in translational regulation and proteostasis, among others, by interacting with proteasome components. While the role of *Pf*SAMS for protein degradation in malaria parasites has so far not been investigated, several studies in other eukaryotes point to a direct link of SAMS in regulating proteasome function via subunit methylation [90]. In other eukaryotes, SAM further appears to directly regulate the mTORC1 pathway by binding to the regulator SAMTOR, which is then unable to inhibit TORC1 signaling [91]. Hence, in the presence of methionine and thus SAM, mTORC1 is active and promotes translation, while during methionine and SAM deficiency, SAMTOR is free to inhibit mTORC1 and block translation. While malaria parasites have lost most of the TORC components through genomic reduction, including mTOR, mLST8, Raptor, and Rictor [92,93,94], the parasites are able to control protein synthesis via various mechanisms of translational regulation [95]. The interaction of *Pf*SAMS with translational elongation factors and ribosome components suggests its direct involvement in these processes, for example, by direct methylation of ribosomal RNA, as has been reported in other organisms [96,97,98].

We also identified the single bifunctional enzyme AdoMetDC/ODC, which is the rate-limiting enzyme in polyamine biosynthesis, in our interaction network, indicating the involvement of *Pf*SAMS in this process. AdoMetDC/ODC has been shown to be important in male gametocyte development and transmission in *P. yoelii* [99]. Its inhibition in *P. falciparum* in vitro causes cytostatic arrest in the trophozoite stage of the asexual blood cycle, but it does not cure *P. berghei* infected mice in vivo [100,101,102]. The co-inhibition of both catalytic sides of the enzyme in *P. falciparum* results in a generalized transcriptional arrest due to polyamine depletion as well as perturbation-specific compensatory transcriptional responses, including the decreased abundance of *pfsams* transcript [103]. Additionally, other enzymes involved in polyamine biosynthesis such as arginase and spermidine synthase were identified by us, further strengthening the important role of *Pf*SAMS in polyamine synthesis.

## 5. Conclusions

Our combined data confirm the vital role of the single enzyme *Pf*SAMS, which generates the vital methyl donor SAM and thus represents a bottleneck of the parasite life cycle for the asexual and sexual blood stages of *P. falciparum*. We show that *Pf*SAMS is crucial for the parasite through its involvement in a complex network with other proteins of various functions. Our interaction network analyses confirm the link of *Pf*SAMS to epigenetic control via methylation of histones, DNA, and RNA, but also due to potential direct interactions with transcription factors. Our data further point to important functions of *Pf*SAMS in PC and polyamine synthesis and in translational control as well as regulation of the proteasome. These newly identified important roles of *Pf*SAMS for parasite viability emphasize the enzyme as a promising antimalarial drug target.

## Data Availability

The mass spectrometry proteomics data have been deposited to the ProteomeXchange Consortium (http://proteomecentral.proteomexchange.org; accessed on 13 July 2022) via the jPOST partner repository [73] with the dataset identifiers PXD034111 (ProteomeXchange) and JPST001602 (jPOST).

## Supplementary Materials

The following supporting information can be downloaded at: https://www.mdpi.com/article/10.3390/microorganisms10071419/s1, Figure S1. Generation and verification of the *Pf*SAMS-HA-KD parasite line; Figure S2. Immunofluorescence control for *Pf*SAMS-HA immunolabeling; Figure S3. The effect of *Pf*SAMS-HA deficiency on asexual blood-stage development; Figure S4. Verification of the *Pf*SAMS-GFP-BirA expression lines; Figure S5. Localization of *Pf*SAMS-GFP-BirA and biotinylated proteins in the blood stages of the *Pf*SAMS-GFP-BirA transfectant lines; Figure S6. String network scheme depicting selected interactors of *Pf*SAMS; Figure S7. Distribution of *Pf*SAMS interactors according to the log2 ratio and functional prediction; Figure S8. Distribution of *Pf*SAMS interactors with enzymatic functions according to the log2 ratio. Table S1. List of primers; Table S2. Combined list of the putative 1114 interactors of *Pf*SAMS; Table S3. Summary of the GO enrichment analysis; Table S4. List of interactors sorted by cluster; Table S5. Selected interactors of *Pf*SAMS sorted by function.
