# Supplementary material for: Plasmodium falciparum S-Adenosylmethionine Synthetase Is Essential for Parasite Survival through a Complex Interaction Network with Cytoplasmic and Nuclear Proteins
Source: Microorganisms. 2022 Jul 14;10(7):1419. doi: 10.3390/microorganisms10071419 (PMC9320499; doi:10.3390/microorganisms10071419)
Supplement: Supplementary file 1 [file microorganisms-10-01419-s001.zip › Musabyimana et al-Microorganisms-Table S1.pdf]

Table S1. List of primers.

Function	Name	Sequence 5′-3′
**Oligonucleotides for the generation of *Pf*92 recombinant protein**		
Cloning primers	*Pf*92rp1 FP	GCGTAGGATCCAATTCTTTGTCAGATAGCC
	*Pf*92rp1 RP	TAGGCCTGCAGTTCCTGAATAGGTGGTTG
**Oligonucleotides for the generation and verification of *Pf*SAMS-HA-KD Mutant**		
Cloning Primers	*Pf*SAMS-HA-*glmS* NotI FP	tcctccGCGGCCGCGTGAATGAAGGACACCCAGAT
	*Pf*SAMS-HA-*glmS* XmaI RP	ctttactCCCGGGATTTTTAATGCATTTTTTTCGTGAGAT
Integration Primers	pSLI-*Pf*SAMS 5′ Int *glmS* FP	GAGGAAACTTTTTATTCACATCTGAA
	pSLI-*Pf*SAMS 3′ Int *glmS* RP	CCACATACGCTTAATTATAATATGATG
	pARL-HA-*glmS* FP	GCTTTACACTTTATGCTTCCGGCTC
	pSLI-HA-*glms* RP	TGTCTGTTGTGCCCAGTCAT
**Oligonucleotides for the generation of pARL-*pfama1*-GFP-BirA vector**		
Cloning primers	GFP FP	AATTAATGTACTTGTTATAAATTGTACAAAGGT ACCGGGCCCCCTAGGagtaaaggagaagaacttttcactgg
	GFP RP	ACCACTTCCTGATGTAGATCCACTACCTTTAGCT CTTCCtttgtagagctcatccatgcc
	BirA FP	GAGCTAAAGGTAGTGGATCTACATCAGGAA GTGGTAAAGATAATACAGTACCATTAAAATTAATAGC
	BirA RP	AAATAAGAAAAACGAACATTAAGCTGCCATAT CCCTCGAGttaTTTTTCAGCTGATCTTAATGATATTTCACC
**Oligonucleotides for the generation of pARL-*pffnpa*-GFP-BirA vector**		
Cloning primers	*pffpna*-5′ FP	AAAAAGCGGCCGCCCATACACCAACCAAC
	pffnpa-3′ RP	AAAAAGGTACCATGATTTATACTAAGGATTGG
**Oligonucleotides for the generation and verification of *Pf*SAMS-*pffnpa*-GFP-BirA mutant**		
Cloning primers	*Pf*SAMS-*pffnpa*-GFP-BirA KpnI FP	cttagtataaatcatggtaccATGAGTCAGTTGAAAATTAAAAG
	*Pf*SAMS-*pffnpa*-GFP-BirA AvrII RP	agttcttctcctttactcctaggATTTTTAATGCATTTTTTTCGTG

Diagnostic PCR Primer	*Pf*SAMS-*pffnpa*-GFP-BirA KpnI FP	cttagtataaatcatggtaccATGAGTCAGTTGAAAATTAAAAG
	GFP RP	CAAGTGTTGGCCATGGAA
	Aldolase RT FP	TAGATGGATTAGCAGAAAGATGC
	Aldolase RT RP	TCAAAATCACGCACATCCTG
**Oligonucleotides for the generation and verification of *Pf*SAMS-*pfama1*-GFP-BirA mutant**		
Cloning Primers	*Pf*SAMS-*pfama1*-GFP-BirA ApaI FP	atgcatgggcccATGAGTCAGTTGAAAATTAAAAGAGG
	*Pf*SAMS-*pfama1*-GFP-BirA AvrII RP	atgcatcctaggATTTTTTAATGCATTTTTTTCGTGAGAT
Diagnostic PCR Primers	PfSAMS-*pfama1*-GFP-BirA ApaI FP	atgcatgggcccATGAGTCAGTTGAAAATTAAAAGAGG
	GFP RP	CAAGTGTTGGCCATGGAA
	Aldolase RT FP	TAGATGGATTAGCAGAAAGATGC
	Aldolase RT RP	TCAAAATCACGCACATCCTG

FP, forward primer; RP, reverse primer; RT, reverse transcriptase; restriction sites indicated in small letters
