# Supplementary material for: Plasmodium falciparum S-Adenosylmethionine Synthetase Is Essential for Parasite Survival through a Complex Interaction Network with Cytoplasmic and Nuclear Proteins
Source: Microorganisms. 2022 Jul 14;10(7):1419. doi: 10.3390/microorganisms10071419 (PMC9320499; doi:10.3390/microorganisms10071419)
Supplement: Supplementary file 1 [file microorganisms-10-01419-s001.zip › Musabyimana et al-Microorganisms-Table S5.pdf]

Table S5. Selected interactors of *Pf*SAMS sorted by function.

Biological function	PlasmoDB ID	Annotation	Log2 ratio
**Polyamine biosynthesis and methionine metabolism**	PF3D7_0922200	**S-adenosylmethionine synthetase (SAMS)**	8.4664
	PF3D7_1033100	S-adenosylmethionine decarboxylase/ornithine decarboxylase (AdoMetDC/ODC)	4.6214
	PF3D7_0906500	Arginase	3.6447
	PF3D7_1129000	Spermidine synthase (SpdSyn)	3.0292
	PF3D7_1034900	Methionine--tRNA ligase (MRSCyt)	3.5505
	PF3D7_1015300	Methionine aminopeptidase 1b, putative (METAP1b)	27.0050
	PF3D7_1434600	Methionine aminopeptidase 2 (METAP2)	3.3955
	PF3D7_0524900	S-adenosyl-L-methionine-dependent tRNA 4-demethylwyosine synthase, putative (TYW1)	24.8037
**Methyltransferases**	PF3D7_1343000	Phosphoethanolamine N-methyltransferase (PMT)	2.3969
	PF3D7_0811500	Histone-arginine methyltransferase (CARM1)	29.8438
	PF3D7_1361000	Protein arginine N-methyltransferase 5, putative (PRMT5)	28.8333
	PF3D7_1426200	Protein arginine N-methyltransferase 1 (PRMT1)	4.0960
	PF3D7_0704200	RNA cytosine C(5)-methyltransferase, putative (NSUN1)	27.8855
	PF3D7_1235600	Serine hydroxymethyltransferase (SHMT)	1.4401
	PF3D7_1429400	rRNA (adenosine-2'-O-)-methyltransferase, putative	27.3882
	PF3D7_1407100	rRNA 2'-O-methyltransferase fibrillarin, putative	2.5999
	PF3D7_0629700	SET domain protein, putative (SET1)	26.5951
	PF3D7_1455200	Methyltransferase AAMT	27.8283
	PF3D7_0912800	tRNA (adenine(58)-N(1))-methyltransferase non-catalytic subunit TRM6, putative (GCD10)	27.1557
	PF3D7_0628600	DNA methyltransferase 1-associated protein 1, putative	27.0066
	PF3D7_1432700	Protein-L-isoaspartate(D-aspartate) O-methyltransferase, putative	26.5256
	PF3D7_0409300	Methyltransferase, putative	26.7827
	PF3D7_1315700	tRNA (adenine(58)-N(1))-methyltransferase catalytic subunit TRM61, putative (GCD14)	26.8921
	PF3D7_1355300	Histone-lysine N-methyltransferase, putative (SET6)	26.5063
	PF3D7_1235500	N6-adenosine-methyltransferase, putative (MA-A70.2)	26.2478
	PF3D7_1115200	Histone-lysine N-methyltransferase SET7 (SET7)	26.0774
	PF3D7_1319300	tRNA (guanine(26)-N(2))-dimethyltransferase, putative (TRM1)	26.4371
	PF3D7_1111000	RNA cytosine C(5)-methyltransferase NSUN2	26.1638
	PF3D7_0908600	Ribosomal RNA methyltransferase, putative	25.5661
	PF3D7_1142700	Methyltransferase, putative	24.9544

	PF3D7_0827800	SET domain protein, putative (SET3)	26.6929
	PF3D7_0508100	SET domain protein, putative (SET9)	25.1961
**Transcription**	PF3D7_0215700	DNA-directed RNA polymerase subunit beta	5.5234
	PF3D7_1304900	Probable DNA-directed RNA polymerase II subunit RPB11	28.9151
	PF3D7_1406200	Transcription elongation factor SPT6, putative	26.8417
	PF3D7_0318200	DNA-directed RNA polymerase subunit	6.0985
	PF3D7_0714500	Transcription elongation factor s-II, putative	3.7430
	PF3D7_0610900	Transcription elongation factor SPT5, putative	26.3238
	PF3D7_1213700	DNA-directed RNA polymerases I, II, and III subunit RPABC3	28.7291
	PF3D7_0923000	DNA-directed RNA polymerase II subunit RPB3, putative	5.5736
	PF3D7_0934100	TFIIH basal transcription factor complex helicase XPD subunit	28.6869
	PF3D7_0110400	DNA-directed RNA polymerase II subunit RPB9, putative	29.8225
	PF3D7_0215700	DNA-directed RNA polymerase subunit beta	5.5234
	PF3D7_1304900	Probable DNA-directed RNA polymerase II subunit RPB11	28.9151
	PF3D7_1406200	Transcription elongation factor SPT6, putative	26.8417
	PF3D7_0318200	DNA-directed RNA polymerase subunit	6.0985
	PF3D7_0714500	Transcription elongation factor s-II, putative	3.7430
	PF3D7_0610900	Transcription elongation factor SPT5, putative	26.3238
	PF3D7_1213700	DNA-directed RNA polymerases I, II, and III subunit RPABC3	28.7291
	PF3D7_0923000	DNA-directed RNA polymerase II subunit RPB3, putative	5.57362
	PF3D7_0934100	TFIIH basal transcription factor complex helicase XPD subunit	28.6869
**AP2 family**	PF3D7_1107800	AP2 domain transcription factor, putative	29.3953
	PF3D7_0604100	AP2 domain transcription factor	27.1111
	PF3D7_0730300	AP2 domain transcription factor AP2-L, putative	27.6788
	PF3D7_0420300	AP2/ERF domain-containing protein	26.3499
	PF3D7_1449500	AP2 domain transcription factor AP2-O5, putative	27.3670
	PF3D7_1466400	AP2 domain transcription factor AP2-EXP	26.7800
	PF3D7_1222600	AP2 domain transcription factor AP2-G	26.2460
	PF3D7_1007700	AP2 domain transcription factor AP2-I	26.5790
	PF3D7_0613800	AP2 domain transcription factor, putative	28.1501
**Translation**	PF3D7_1410600	Eukaryotic translation initiation factor 2 subunit gamma, putative (elF2gamma)	4.1457
	PF3D7_0728000	Eukaryotic translation initiation factor 2 subunit alpha	4.1215
	PF3D7_1206200	Eukaryotic translation initiation factor 3 subunit C, putative (EIF3C)	3.6096

	PF3D7_0315100	Eukaryotic translation initiation factor 4E	4.7842
	PF3D7_0418200	Eukaryotic translation initiation factor 3 subunit M, putative	3.0595
	PF3D7_0517700	Eukaryotic translation initiation factor 3 subunit B, putative	3.3077
	PF3D7_0607000	Translation initiation factor IF-2, putative	4.1469
	PF3D7_0716800	Eukaryotic translation initiation factor 3 subunit I, putative	2.4184
	PF3D7_0815600	Eukaryotic translation initiation factor 3 subunit G, putative	3.0470
	PF3D7_0828500	Translation initiation factor eIF-2B subunit alpha, putative	27.1305
	PF3D7_0907600	Translation initiation factor SUI1, putative	27.6126
	PF3D7_0918300	Eukaryotic translation initiation factor 3 subunit F, putative	3.2194
	PF3D7_1007900	Eukaryotic translation initiation factor 3 subunit D, putative	2.1301
	PF3D7_1010600	Eukaryotic translation initiation factor 2 subunit beta	4.8132
	PF3D7_1013900	Translation initiation factor eIF-2B subunit delta, putative	28.3519
	PF3D7_1019000	Eukaryotic translation initiation factor subunit eIF2A, putative	27.9313
	PF3D7_1112500	RNA transcription, translation and transport factor protein, putative	25.1258
	PF3D7_1123400	Eukaryotic peptide chain release factor GTP-binding subunit, putative	4.3834
	PF3D7_1204300	Eukaryotic translation initiation factor 5A	1.4045
	PF3D7_1212700	Eukaryotic translation initiation factor 3 subunit A, putative	3.1286
	PF3D7_1244800	Translation machinery-associated protein 46, putative	27.3477
	PF3D7_1250600	Translation initiation factor eIF-2B subunit beta, putative	28.7134
	PF3D7_1305300	Translational activator GCN1, putative	4.8953
	PF3D7_1312900	Eukaryotic translation initation factor 4 gamma	2.8445
	PF3D7_1326400	Translation initiation factor eIF-2B subunit gamma, putative	28.9044
	PF3D7_1332800	Eukaryotic translation initiation factor 6, putative	27.1034
	PF3D7_1438000	Eukaryotic translation initiation factor eIF2A, putative	3.01972
**Proteasome**	PF3D7_1368100	26S proteasome regulatory subunit RPN11, putative	4.4773
	PF3D7_0727400	Proteasome subunit alpha type	4.4586
	PF3D7_1402300	26S proteasome regulatory subunit RPN6	4.3866
	PF3D7_1338100	26S proteasome regulatory subunit RPN3, putative	4.5732
	PF3D7_0608500	Proteasome endopeptidase complex	5.2780
	PF3D7_1353900	Proteasome subunit alpha type	5.2052
	PF3D7_0205900	26S proteasome regulatory subunit RPN1, putative	4.0512
	PF3D7_0727400	Proteasome subunit alpha type	4.4586
	PF3D7_1466300	26S proteasome regulatory subunit RPN2, putative	4.3325
	PF3D7_0807500	Proteasome endopeptidase complex	5.3799

PF3D7_1338100	26S proteasome regulatory subunit RPN3, putative	4.5732
PF3D7_0317000	Proteasome subunit alpha type-3, putative	5.6213
PF3D7_0518300	Proteasome subunit beta	5.3307
PF3D7_1017900	26S proteasome regulatory subunit p55, putative	5.0310
PF3D7_0528000	Proteasome maturation factor UMP1, putative	27.7918
PF3D7_0912900	26S proteasome regulatory subunit RPN8, putative	4.9984
PF3D7_1129200	26S proteasome regulatory subunit RPN7, putative	4.4330
PF3D7_1030500	26S proteasome regulatory subunit RPN9, putative	4.5958
PF3D7_0807800	26S proteasome regulatory subunit RPN10, putative	2.2973
PF3D7_0723600	Proteasome assembly chaperone 4, putative	28.5994
PF3D7_0317800	26S proteasome non-ATPase regulatory subunit 9, putative	4.19063
PF3D7_1414000	26S proteasome regulatory subunit RPN13, putative	4.8404
