# Supplementary material for: Plasmodium falciparum S-Adenosylmethionine Synthetase Is Essential for Parasite Survival through a Complex Interaction Network with Cytoplasmic and Nuclear Proteins
Source: Microorganisms. 2022 Jul 14;10(7):1419. doi: 10.3390/microorganisms10071419 (PMC9320499; doi:10.3390/microorganisms10071419)
Supplement: Supplementary file 1 [file microorganisms-10-01419-s001.zip › Musabyimana et al-Microorgranisms-Fig S1.pdf]

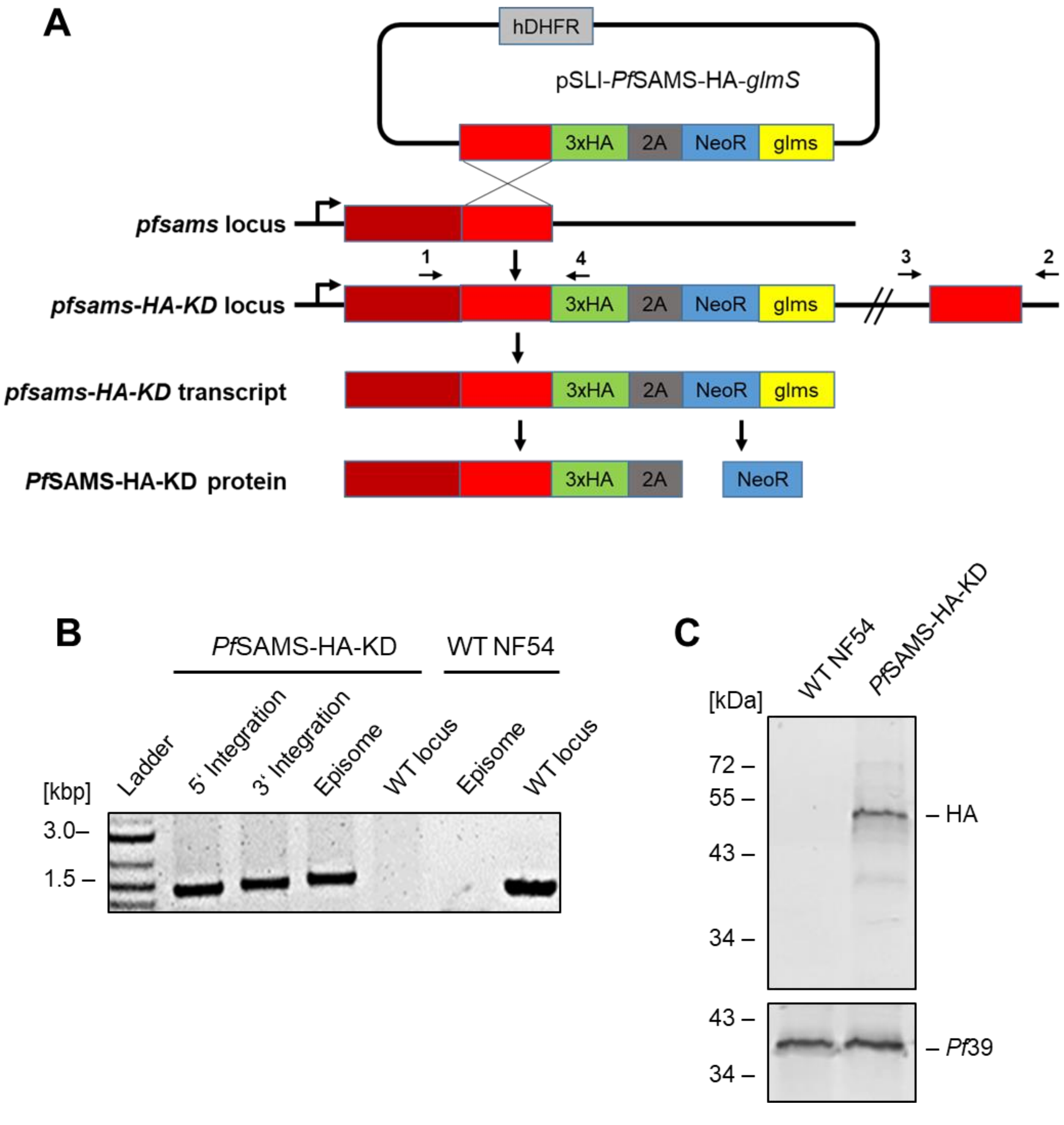

Figure S1. Generation and verification of the *Pf*SAMS-HA-KD parasite line. (A) Schematic depicting the single-crossover homologous recombination strategy for the generation of the *Pf*SAMS-HA-KD line. The coding region of *pfsams* was fused at the 3′-end to a HA-encoding sequence followed by the 2A-skip peptide sequence and the *glmS*-ribozyme sequence. The numbered arrows indicate the positions of primers used to confirm integration of the pSLI-*Pf*SAMS-HA-*glmS* vector. *glmS*, glucosamine-6-phosphate-activated ribozyme; HA, hemagglutinin; hDHFR, human dihydrofolate reductase-encoding gene conferring resistance to WR99210; NeoR, gene conferring resistance to neomycin. (B) Confirmation of gene locus integration of the pSLI-*Pf*SAMS-HA-*glmS* vector. Diagnostic PCR demonstrates successful 5′ (1,412 bp; primers 1 and 4) and 3′ (1,471 bp; primers 2 und 3) integration. As a control, WT NF54 gDNA was used, demonstrating the original gene locus (1,368 bp; primers 1 and 2). Episomal DNA was further detected (1,251 bp; primers 3 and 4). (C) Detection of *Pf*SAMS-HA in the *Pf*SAMS-HA-KD parasite line. Mixed asexual blood stage lysates from the *Pf*SAMS-HA-KD parasite line as well as from WT NF54 for control were immunoblotted with rat anti-HA antibody to detect *Pf*SAMS-HA (~50 kDa); immunoblotting with rabbit anti-*Pf*39 antisera (39 kDa) was used for loading control.
