# Supplementary material for: Plasmodium falciparum S-Adenosylmethionine Synthetase Is Essential for Parasite Survival through a Complex Interaction Network with Cytoplasmic and Nuclear Proteins
Source: Microorganisms. 2022 Jul 14;10(7):1419. doi: 10.3390/microorganisms10071419 (PMC9320499; doi:10.3390/microorganisms10071419)
Supplement: Supplementary file 1 [file microorganisms-10-01419-s001.zip › Musabyimana et al-Microorgranisms-Fig S2.pdf]

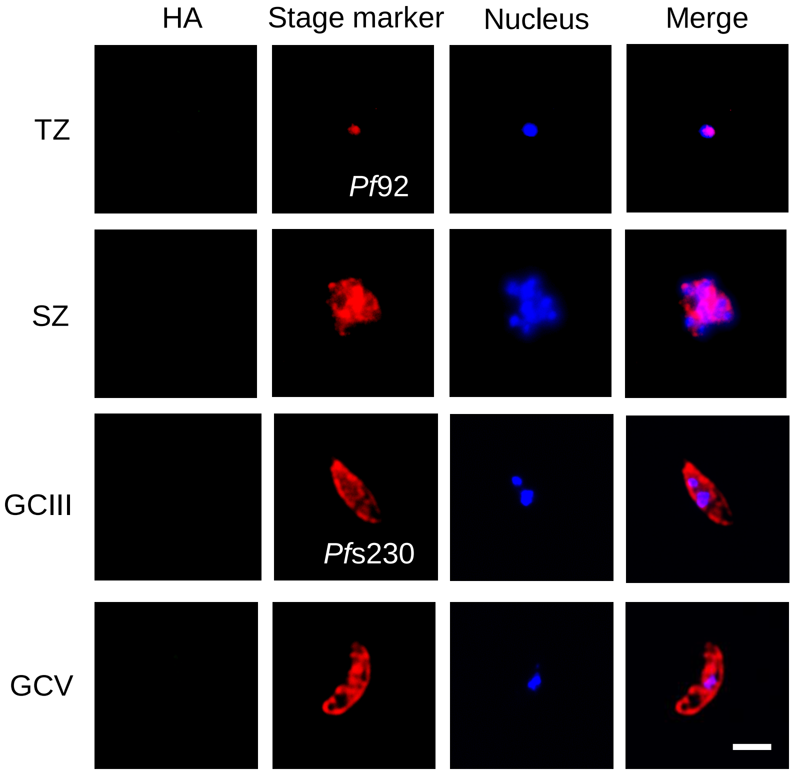

Figure S2. Immunofluorescence control for the *Pf*SAMS-HA immunolabeling. Immunofluorescence assays were employed, using rat anti-HA antibody to detect *Pf*SAMS-HA in trophozoites (TZ), schizonts (SZ) and gametocytes (GC) of stages III and V (green) of WT NF54. Counterlabeling of the asexual blood stages was done with mouse anti-*Pf*92 antisera and of gametocytes with rabbit anti-*Pf*s230 antisera (red). The nuclei were labeled with Hoechst 33342 nuclear stain (blue). Bar; 5 µm. The results are representative of three independent experiments.
