# Supplementary material for: Plasmodium falciparum S-Adenosylmethionine Synthetase Is Essential for Parasite Survival through a Complex Interaction Network with Cytoplasmic and Nuclear Proteins
Source: Microorganisms. 2022 Jul 14;10(7):1419. doi: 10.3390/microorganisms10071419 (PMC9320499; doi:10.3390/microorganisms10071419)
Supplement: Supplementary file 1 [file microorganisms-10-01419-s001.zip › Musabyimana et al-Microorgranisms-Fig S3.pdf]

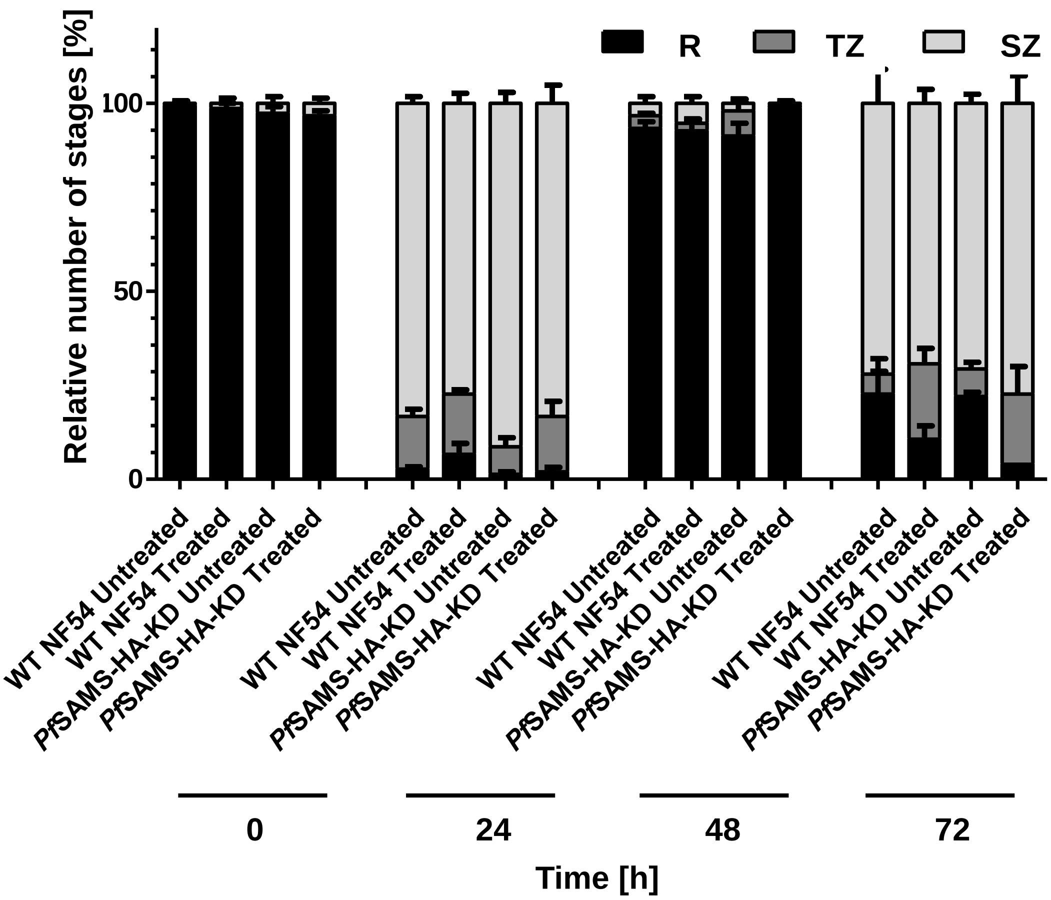

Figure S3. The effect of *Pf*SAMS-HA deficiency on asexual blood stage development. Synchronized ring stage cultures of WT NF54 and the *Pf*SAMS-HA-KD parasite line were treated with 5 mM GlcN at a starting parasitemia of 0.25% and the cultures were maintained in cell culture medium with GlcN over a time-period of 72 h. Rings (R), trophozoites (TZ), and schizonts (SZ) were determined in a total number of 100 iRBCs in triplicate every 24 h via Giemsa-stained blood smears. The results are representative of two independent experiments.
