# Supplementary material for: Plasmodium falciparum S-Adenosylmethionine Synthetase Is Essential for Parasite Survival through a Complex Interaction Network with Cytoplasmic and Nuclear Proteins
Source: Microorganisms. 2022 Jul 14;10(7):1419. doi: 10.3390/microorganisms10071419 (PMC9320499; doi:10.3390/microorganisms10071419)
Supplement: Supplementary file 1 [file microorganisms-10-01419-s001.zip › Musabyimana et al-Microorgranisms-Fig S4.pdf]

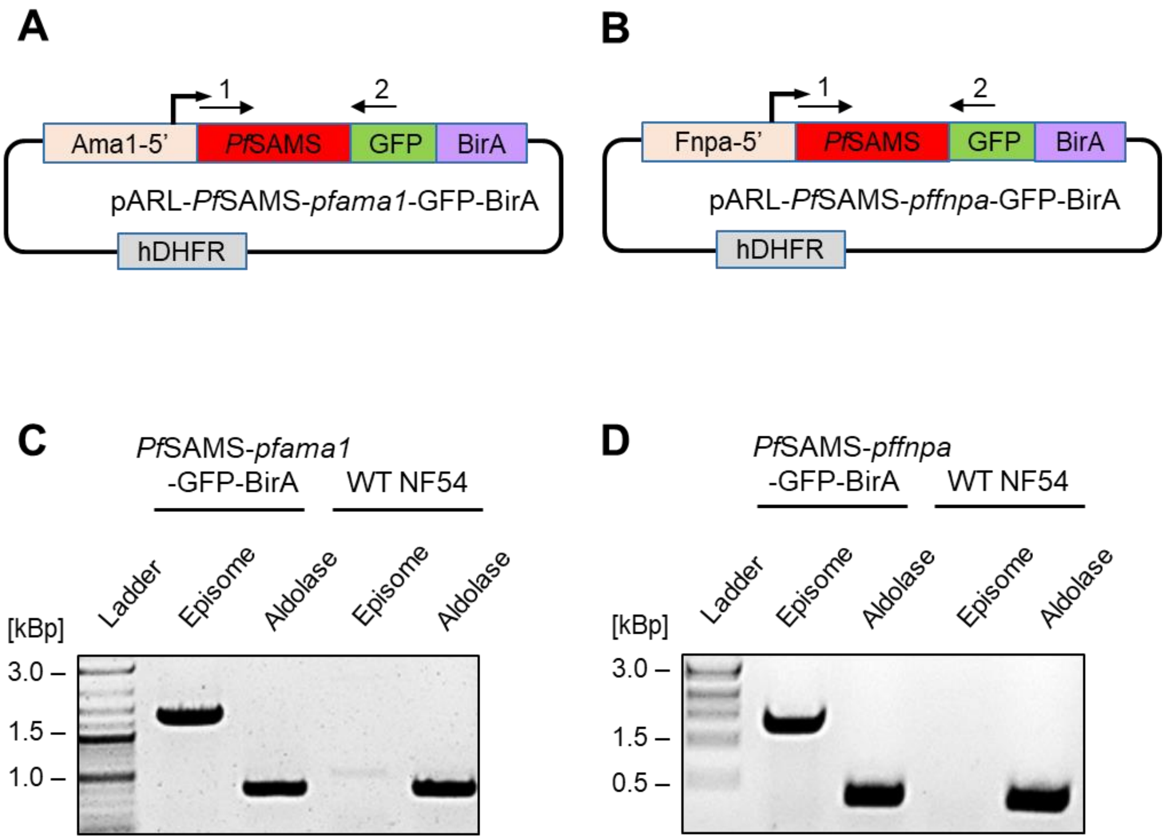


**Figure S4. Verification of the *Pf*SAMS-GFP-BirA expression lines.** (A, B) Schematics depicting the vectors pARL-*pfama1*-*Pf*SAMS-BirA (A) and pARL-*pffnpa*-*Pf*SAMS-BirA (B). BirA, *E. coli* biotin ligase; GFP, green fluorescent protein; hDHFR, human dihydrofolate reductase-encoding gene conferring resistance to WR99210. (D, E) Detection of episomal uptake in the transfectant parasites. Diagnostic PCR was employed to detect the presence of vectors pARL-*Pf*SAMS-*pfama1*-GFP-BirA (C) and pARL-*Pf*SAMS-*pffnpa*-GFP-BirA (D) (1,423 bp; primers 1 and 2) in the transfectant lines, but not the WT NF54. Primers specific for the gene encoding plasmodial aldolase (378 bp) were used as control.
