# Supplementary material for: Plasmodium falciparum S-Adenosylmethionine Synthetase Is Essential for Parasite Survival through a Complex Interaction Network with Cytoplasmic and Nuclear Proteins
Source: Microorganisms. 2022 Jul 14;10(7):1419. doi: 10.3390/microorganisms10071419 (PMC9320499; doi:10.3390/microorganisms10071419)
Supplement: Supplementary file 1 [file microorganisms-10-01419-s001.zip › Musabyimana et al-Microorgranisms-Fig S5.pdf]

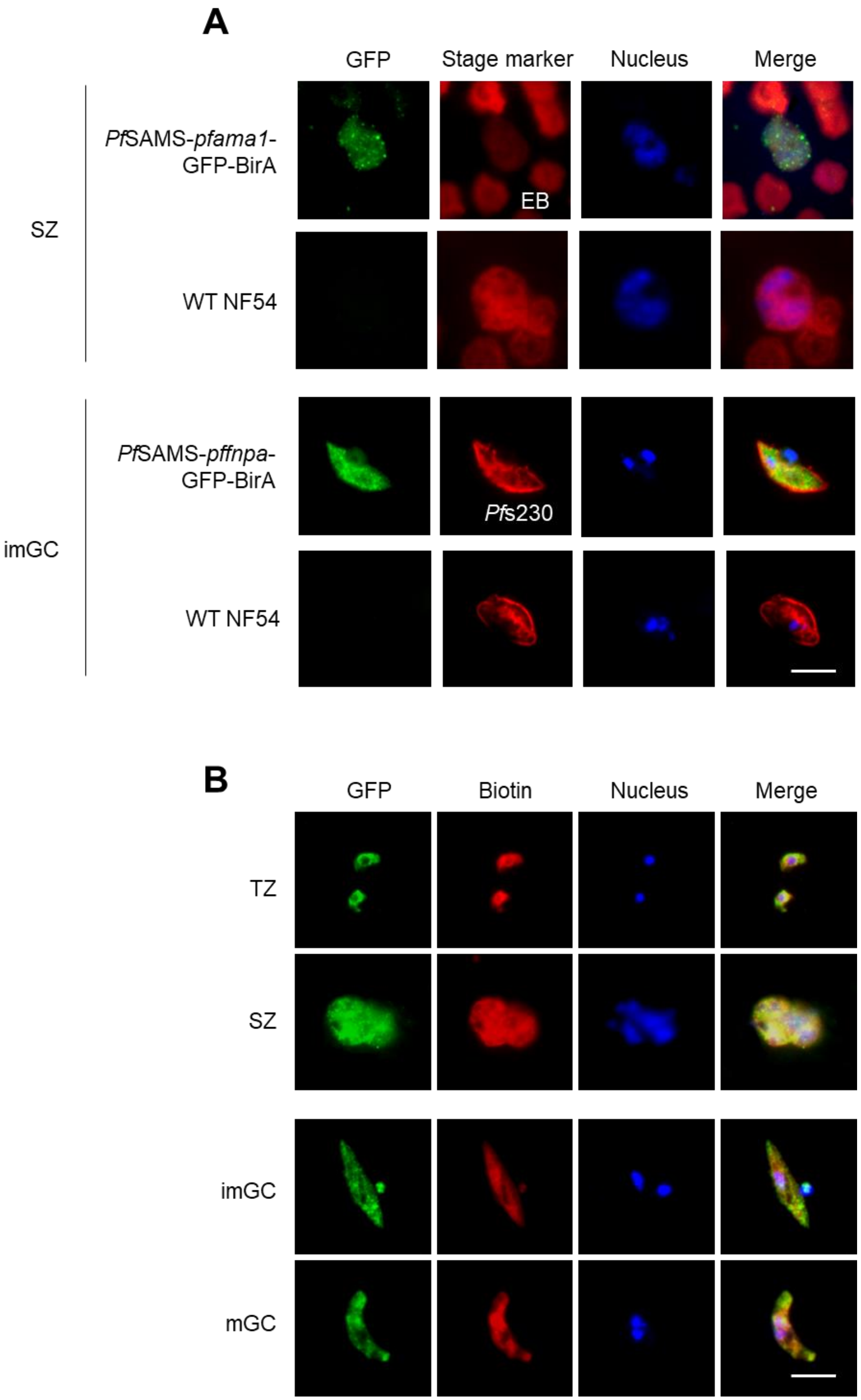

Figure S5. Localization of *Pf*SAMS-GFP-BirA and biotinylated proteins in the blood stages of the *Pf*SAMS-GFP-BirA transfectant lines. (A) Labeling of *Pf*SAMS-GFP-BirA in blood stage parasites. Immunofluorescence assays were employed, using asexual blood stages of the *Pf*SAMS-*pfama1*-GFP-BirA line and gametocytes of the *Pf*SAMS-*pffnpa*-GFP-BirA line. Mouse antibodies directed against GFP were used to detect *Pf*SAMS-GFP-BirA in schizonts (SZ) and immature gametocytes (imGC) (green). Counterlabeling of the asexual blood stages was done with Evans Blue (EB) stain and of gametocytes with rabbit anti-*Pf*s230 antisera (red). The nuclei were labeled with Hoechst 33342 nuclear stain (blue). WT NF54 parasites were used for negative control. (B) Co-localization of *Pf*SAMS-GFP-BirA and biotinylated proteins. Asexual blood stages of the *Pf*SAMS-*pfama1*-GFP-BirA line and gametocytes of the *Pf*SAMS-*pffnpa*-GFP-BirA line were treated with 50 µM biotin for 24 h and subjected to immunofluorescence assays as described above. Biotinylated proteins were immunolabeled using fluorophore-conjugated streptavidin (red). mGC, mature gametocyte; TZ, trophozoite. Bar; 5 µm. The results (A, B) are representative of two independent experiments.
