# Supplementary material for: Plasmodium falciparum S-Adenosylmethionine Synthetase Is Essential for Parasite Survival through a Complex Interaction Network with Cytoplasmic and Nuclear Proteins
Source: Microorganisms. 2022 Jul 14;10(7):1419. doi: 10.3390/microorganisms10071419 (PMC9320499; doi:10.3390/microorganisms10071419)
Supplement: Supplementary file 1 [file microorganisms-10-01419-s001.zip › Musabyimana et al-Microorgranisms-Fig S6.pdf]

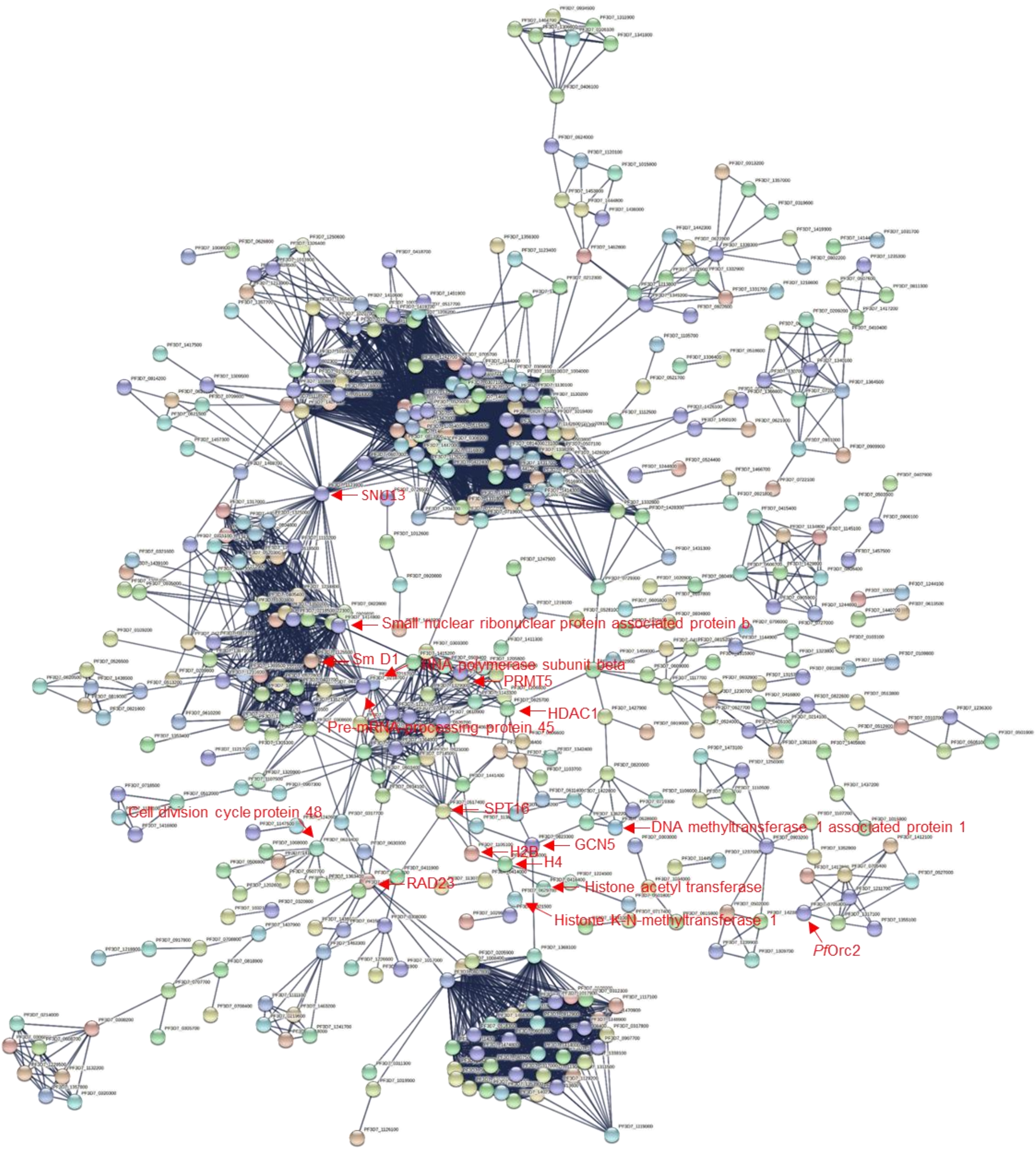

Fig. S6. Network analysis scheme depicting selected interactors of *Pf*SAMS. A list of 1,114 query proteins was evaluated for potential interactions, using the String database. A network of the potential *Pf*SAMS interactors was predicted with a confidence level of 0.009. A Markov Clustering (MCL) algorithm was used to describe possible clusters with an inflation parameter of 3. Based on the physical interaction among only the query proteins, different clusters of interactors were identified.
