# Supplementary material for: Plasmodium falciparum S-Adenosylmethionine Synthetase Is Essential for Parasite Survival through a Complex Interaction Network with Cytoplasmic and Nuclear Proteins
Source: Microorganisms. 2022 Jul 14;10(7):1419. doi: 10.3390/microorganisms10071419 (PMC9320499; doi:10.3390/microorganisms10071419)
Supplement: Supplementary file 1 [file microorganisms-10-01419-s001.zip › Musabyimana et al-Microorgranisms-Fig S7.pdf]

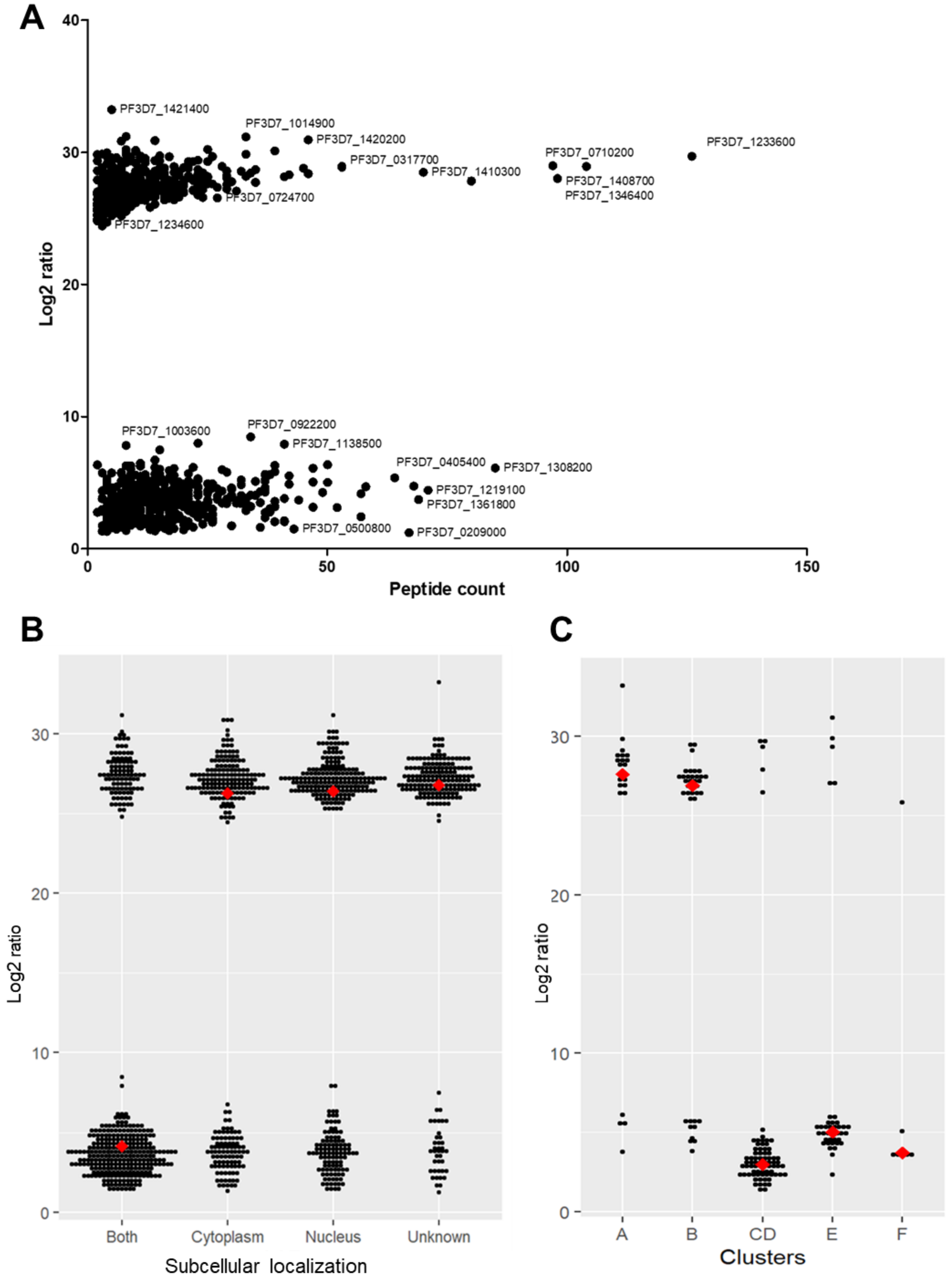

Figure S7. Distribution of *Pf*SAMS interactors according to log2 ratio and functional prediction. The 1,114 identified interaction partners were plotted based on the peptide count and the log2 ratio. (A) Dotplot graph depicting the interactors aggregating in two distinct groups based on their log2 ratio and peptide count. Selected proteins representing each group are labeled with their PlasmoDB gene IDs. (B) Dotplot graph depicting the subcellular localization of the interactors of both groups. The GO categories "Nucleus" (GO:0005634) and "Cytoplasm" (GO:0005737) were considered. (C) Dotplot graph comparing the clusters A-F, as identified by STRING analysis, based on the log2 ratio. The six clusters are defined in Fig. 6. The red diamond (B, C) represents the median of each group.
