# Supplementary material for: Plasmodium falciparum S-Adenosylmethionine Synthetase Is Essential for Parasite Survival through a Complex Interaction Network with Cytoplasmic and Nuclear Proteins
Source: Microorganisms. 2022 Jul 14;10(7):1419. doi: 10.3390/microorganisms10071419 (PMC9320499; doi:10.3390/microorganisms10071419)
Supplement: Supplementary file 1 [file microorganisms-10-01419-s001.zip › Musabyimana et al-Microorgranisms-Fig S8.pdf]

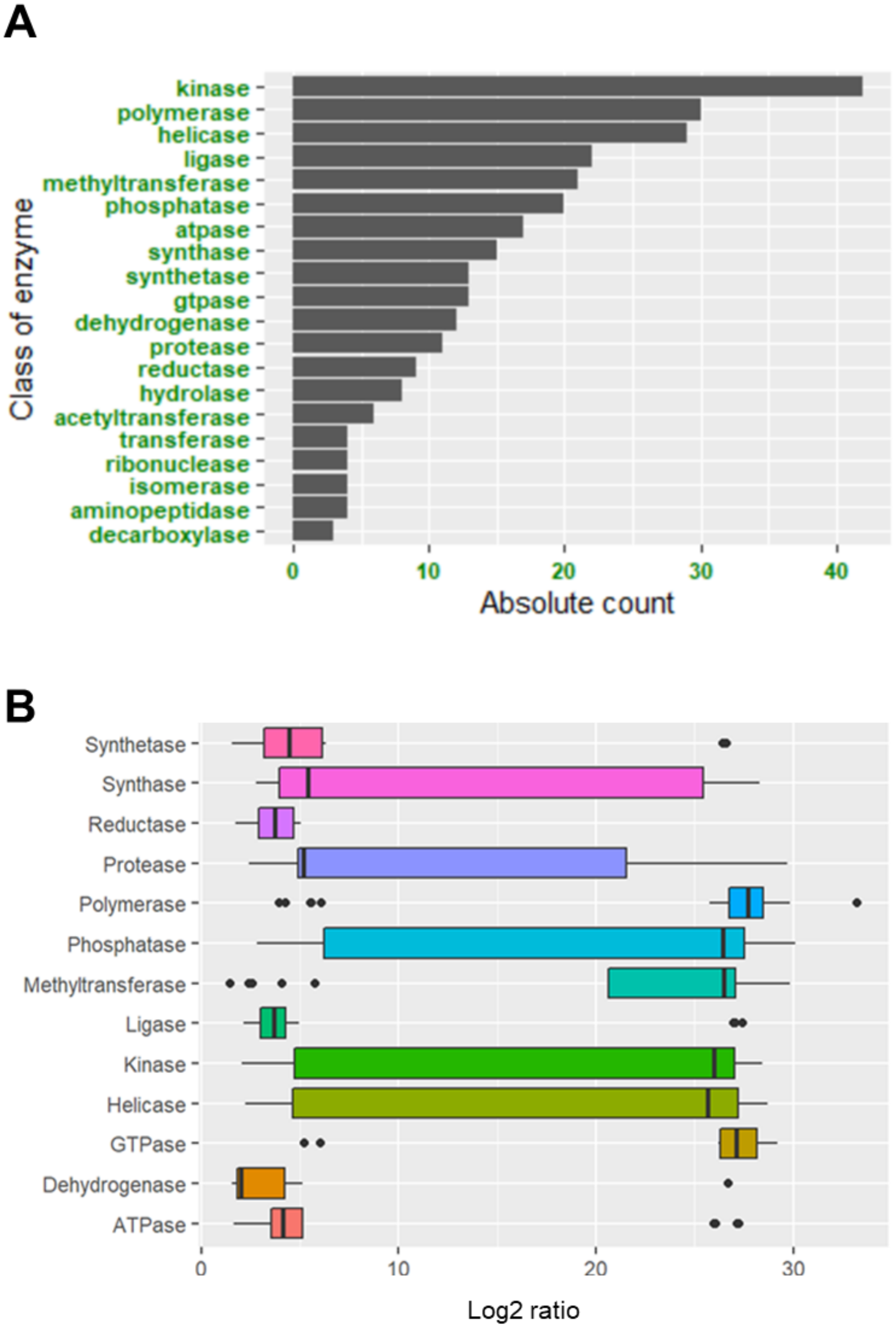

Figure S8. Distribution of *Pf*SAMS interactors with enzymatic functions according to the log2 ratio. The 1,114 identified interaction partners with enzymatic function were plotted based on the peptide count and the log2 ratio. (A) Bar chart showing the classes of enzymes identified in the groups of *Pf*SAMS interactors per absolute count. Class names were obtained by text mining of *Pf*SAMS interactors' fasta headers acquired from mass spectrometry analysis. (E) Box plot showing the classes of enzymes as identified in (D), based on the log2 ratio. The line inside each boxplot shows the median of each class.
